# Duplicate Gene Divergence by Changes in MicroRNA Binding Sites in *Arabidopsis* and *Brassica*

**DOI:** 10.1093/gbe/evv023

**Published:** 2015-02-02

**Authors:** Sishuo Wang, Keith L. Adams

**Affiliations:** Department of Botany, University of British Columbia, Vancouver, British Columbia, Canada

**Keywords:** gene duplication, whole-genome duplication, microRNAs, gene regulation, tandem duplicates, microRNA binding sites

## Abstract

Gene duplication provides large numbers of new genes that can lead to the evolution of new functions. Duplicated genes can diverge by changes in sequences, expression patterns, and functions. MicroRNAs play an important role in the regulation of gene expression in many eukaryotes. After duplication, two paralogs may diverge in their microRNA binding sites, which might impact their expression and function. Little is known about conservation and divergence of microRNA binding sites in duplicated genes in plants. We analyzed microRNA binding sites in duplicated genes in *Arabidopsis thaliana* and *Brassica rapa.* We found that duplicates are more often targeted by microRNAs than singletons. The vast majority of duplicated genes in *A. thaliana* with microRNA binding sites show divergence in those sites between paralogs. Analysis of microRNA binding sites in genes derived from the ancient whole-genome triplication in *B. rapa* also revealed extensive divergence. Paralog pairs with divergent microRNA binding sites show more divergence in expression patterns compared with paralog pairs with the same microRNA binding sites in *Arabidopsis*. Close to half of the cases of binding site divergence are caused by microRNAs that are specific to the *Arabidopsis* genus, indicating evolutionarily recent gain of binding sites after target gene duplication. We also show rapid evolution of microRNA binding sites in a jacalin gene family. Our analyses reveal a dynamic process of changes in microRNA binding sites after gene duplication in *Arabidopsis* and highlight the role of microRNA regulation in the divergence and contrasting evolutionary fates of duplicated genes.

## Introduction

Gene duplication is a major mechanism of new gene creation that has led to the evolution of new gene functions (reviewed in Zhang 2003; Flagel and Wendel 2009). Duplicated genes can be generated by whole-genome duplication (WGD), tandem duplication (TD), retroposition, and other mechanisms. After gene duplication, paralogs may have multiple different fates (reviewed in Semon and Wolfe 2007; Innan and Kondrashov 2010). Many paralogs show divergence in gene structure, expression pattern, and function. The functions of duplicated genes can diverge by the acquisition of new function, neofunctionalization, or partitioning of ancestral function, subfunctionalization (Hughes 1994; Force et al. 1999). Expression patterns of duplicated genes can diverge by changes in gene regulation, including gain of a new expression pattern relative to the ancestral state or partitioning of an ancestral expression pattern between the duplicates, also referred to as neofunctionalization and subfunctionalization, respectively (Force et al. 1999). Functional and expression divergence are widely regarded as important mechanisms for the retention of duplicated genes.

MicroRNAs (miRNAs), a kind of short noncoding RNA (Cuperus et al. 2011), play important roles in the regulation of gene expression at the posttranscriptional level by transcript degradation or suppression of translation (Bonnet et al. 2006; Li and Mao 2007; Meng et al. 2011; Takuno and Innan 2011) and may provide a dynamic way to regulate gene expression in many eukaryotes (Berezikov 2011; Rogers and Chen 2013). In plants, gene silencing mediated by miRNAs is an important mechanism in regulating some developmental processes (Chen 2009; Rubio-Somoza and Weigel 2011) and the response to stress (Sunkar et al. 2012), among other functions. Some of the most common miRNA targets in plants include transcription factors and F-box domain-containing proteins (Rhoades et al. 2002; Jones-Rhoades et al. 2006).

Although several of the proteins in miRNA regulation systems are shared by a wide range of plants and animals, the molecular mechanism of the action of miRNAs has been shown to be different between animals and plants in many ways (Chen and Rajewsky 2007; Axtell and Bowman 2008; Voinnet 2009). One distinction is that miRNAs often tend to target protein-coding regions of mRNAs in plants but 3′-untranslated regions (UTRs) in animals (Filipowicz et al. 2008), implying that in plants the miRNA binding sites of protein-coding genes may be under stronger selective pressure and evolve more slowly (Chen and Rajewsky 2007; Guo et al. 2008). Another distinction lies in the mechanism of target recognition. In plants, the recognition of target sites often requires relatively extensive complementarity between miRNAs and target sites (Iwakawa and Tomari 2013; Rogers and Chen 2013). In animals, miRNA-target interactions are more tolerant to mismatches in pairing (Zeng and Cullen 2004; Bartel 2009). The high fidelity of pairing between miRNAs and targets makes the prediction of target genes and their miRNA binding sites easier and more reliable in plants (Rhoades et al. 2002; Jones-Rhoades and Bartel 2004).

A few studies have examined miRNA-target interactions in duplicated genes. Li et al. (2008) found that miRNAs appear to preferentially regulate duplicated genes over singletons in mammals, based on miRNA binding site prediction results. This finding was further supported by another study where genes localized in CNV (copy number variation) regions were shown to have more miRNA-predicted targets in human (Felekkis et al. 2011). In *Arabidopsis*, Takuno and Innan (2008) showed a negative correlation between the copy numbers of miRNAs and the size of the gene families they regulate. Despite these studies, a genome-wide analysis characterizing the evolution of miRNA regulation in duplicated gene pairs has not been reported. Divergence in miRNA regulation between duplicated genes may be an important mechanism of divergence in expression and function.

We conducted a systematic analysis of the evolution of miRNA binding sites after gene duplication using duplicated genes in Brassicaceae, with a focus on *Arabidopsis thaliana* because of the large number of identified miRNAs and experimentally verified miRNA-target interactions in that species. We analyzed whole-genome duplicates from the alpha-WGD in the *Arabidopsis* lineage, tandem duplicates, and other types of duplicates. We also analyzed genes in *Brassica rapa* generated by the whole-genome triplication (WGT) in its lineage as another and more recent polyploidy event.

## Materials and Methods

### Duplicate Gene Data Sets

Genes from *A. thaliana* used in this study were retrieved from TAIR (Lamesch et al. 2011). Sequences annotated as transposable elements were eliminated from the analyses based on TAIR annotation. An all-against-all BLASTP search was performed to identify duplicate and singleton genes in *A. thaliana*. Sequences with *E* values less than 1e-10 (as used for defining duplicates in Casneuf et al. 2006; He and Zhang 2006; Su et al. 2006; Yang and Gaut 2011) and sequence coverage above 50% were defined as duplicates, and those having no nonself hits with *E* values less than 1e-3 were considered to be singletons (as in Amoutzias et al. 2010). Genes encoded by the mitochondrial genome or chloroplast genome were removed.

Duplicates derived from the alpha-WGD in *A. thaliana* were from the Blanc and Wolfe data set (Blanc et al. 2003) which contains 2,584 pairs of duplicates generated by the most recent WGD event (alpha-WGD) at the base of the Brassicaceae family. Also 1,096 pairs of tandem duplicate pairs were obtained from Haberer et al. (2004). In addition we identified 3,178 pairs of other types of duplicates, defined as those with best reciprocal hits and not overlapping WGD duplicates and tandem duplicates. In total, a set of 6,858 pairs of paralogous gene pairs from *A. thaliana* generated by different mechanisms was analyzed. Paralogous genes derived from the *Brassica* lineage-specific genome triplication and their syntenic information were obtained from Cheng et al. (2012).

### miRNA Data Sets

miRNA sequences from *A. thaliana* and *B. rapa* were downloaded from miRBase (Griffiths-Jones et al. 2006), a widely used database for miRNA resources which includes a large number of experimentally verified miRNAs in a wide range of species. The mature miRNA sequences were used to predict miRNA binding sites.

To define young and ancient miRNAs, we performed a BLASTN search against the genomes of 23 plant species (see supplementary table S4, Supplementary Material online, for the full list). Young miRNAs were defined as those with no BLAST hits outside of the *Arabidopsis* genus at the *E* value cutoff of 1e-10, sequence coverage above 50%, and in addition without homologs outside of the *Arabidopsis* genus based on the annotation of miRBase. Other miRNAs were defined as ancient. Lists of young and ancient miRNAs are in supplementary table S4, Supplementary Material online.

### Analysis of miRNA Target Genes

Computational methods have also been shown to be powerful tools in prediction of miRNA targets in plants (Jones-Rhoades and Bartel 2004; Wang et al. 2004; Chen et al. 2010). Many prediction tools have been developed for plant-specific miRNA target gene prediction in the past 5 years (Dai et al. 2011). In this study, we used the following three plant-specific miRNA binding sites prediction methods: psRNAtarget (Dai and Zhao 2011), Tapir (Bonnet et al. 2010), and the miRNA target prediction tool implemented in UEA sRNA workbench (Stocks et al. 2012) to predict potential miRNA targets. All of the three prediction tools are thought to be powerful tools in miRNA-target interaction predictions specific to plants and have been widely utilized (Jeong et al. 2011; Shivaprasad et al. 2012; Wang et al. 2012; McHale et al. 2013; Weiberg et al. 2013). The default cutoff value of the number of mismatched base pairs was used for each program: 3 for psRNAtarget, 3.5 for TAPIR, and 3 for sUEA. Each G:U and non-G:U mismatch is counted as 0.5 points and 1 point, respectively (Jones-Rhoades and Bartel 2004; Schwab et al. 2005; Lu et al. 2008). It is thought that the combination of the use of multiple methods would help to decrease the false positive rate of prediction methods and get more accurate results compared with using a single prediction method (Dai et al. 2011; Ding et al. 2012). Thus in this study we define a positive miRNA-target interaction when it is predicted by at least two of the three prediction programs in order to get predicted miRNA targets with higher confidence. The prediction data set is listed in supplementary table S2, Supplementary Material online. When comparing the prediction data set with the experimental data set, we found that 112 of the 156 experimentally verified miRNA-target interactions were included in the prediction data set, which is 72% overlap between the two data sets.

Experimentally verified miRNA targets of *A. thaliana* were manually collected based on the combination of multiple publications and miRNA target databases (Sun et al. 2013; Hsu et al. 2014). The experimental data include miRNA-target interaction results from both degradome sequencing and low-throughput technologies. The final data set contains 156 experimentally verified miRNA-target interactions in 145 protein-coding genes (supplementary table S2, Supplementary Material online).

### Sequence and Expression Analyses

The alignment of paralogous genes was done using MUSCLE v3.8.31(Edgar 2004). The Yn00 program implemented in PAML (version 4.7) (Yang 2007) was used to calculate Ka/Ks values of duplicated genes. Normalized expression data from 63 different organs and developmental stages of *A. thaliana* were collected from AtGenExpress (http://arabidopsis.org/servlets/TairObject?type=expression_set&id=1006710873 last accessed February 13, 2015) and were used to calculate the Pearson correlation coefficient of expression patterns between duplicates. Jacalin domain containing proteins were identified by using hmmscan (Eddy 1998) with a cutoff *E* value of 1e-10. The best-fit substitution model used in phylogenetic reconstruction was determined as WAG+G+F+I (Whelan and Goldman 2001) using Prottest (Darriba et al. 2011). Phylogenetic trees were constructed with RAxML v7.3.9 (Stamatakis 2006) and 1,000 bootstrap replicates were performed to obtain the support value for each node of the tree. The final tree was visualized using FigTree v1.3.1. The phylogenetic tree and the alignment (supplementary fig. S2, Supplementary Material online) of jacalin domain containing proteins in *A. thaliana* were deposited at TreeBase (Morell 1996) under the accession S16068.

Sequence format processing was done with scripts written in Perl and Ruby (Goto et al. 2010) (available upon request).

## Results

### Duplicates Are More Often Targeted by miRNAs than Singletons

**Fig. 1.—:**
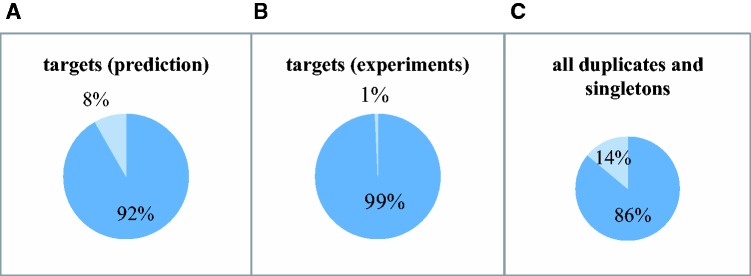
Duplicated genes are more likely to be targeted by miRNAs than singletons. The proportions of duplicates and singletons among all miRNA targets based on binding site prediction data set (*A*) and experimental data set (*B*) are indicated. The proportions of all duplicates and singletons in the genome are shown in (*C*). Lighter and darker portions of the pie charts represent singletons and duplicates, respectively.

To determine whether duplicated genes or singletons in *A. thaliana* are more likely to be under miRNA regulation, we assembled defined sets of 22,054 duplicates and 3,520 singletons (see Materials and Methods) listed in supplementary table S1, Supplementary Material online. We manually collected experimentally verified miRNA targets in *A. thaliana* from different publications and databases (see Materials and Methods). The final data set of known miRNA targets contains 145 protein-coding genes with 156 miRNA-target interactions. Surprisingly, only one of them was a singleton (fig. 1*B*). We found that 0.6% of duplicates and 0.03% of singletons are miRNA targets. Overall the analyses indicate that duplicated genes are indeed more likely to be targeted by miRNAs than singletons in *A. thaliana* based on the experimental data set (*P* < 1e-4, chi-square test).

It is possible that duplicated genes might be overrepresented in the experimentally verified data set for miRNA-target interaction because they happened to be more highly studied than singletons. Also, all possible miRNA-target interactions in *A. thaliana* have not been experimentally identified. To further test whether miRNA targets are indeed more enriched in duplicates than in singletons, we analyzed all possible miRNA-target interactions genome-wide using prediction methods. Three plant-specific prediction methods: UEA sRNA (Stocks et al. 2012), psRNAtarget (Dai and Zhao 2011), and TAPIR (Bonnet et al. 2010) were used in this study. Given the inaccuracy caused by individual prediction programs, only those genes predicted to be the targets by at least two of three programs are considered as potential targets. The combination of different computational tools is thought to be able to minimize the negative impact of using only one program to predict miRNA targets (Dai et al. 2011; Ding et al. 2012). Based on this criterion, 1,210 miRNA-target interactions including 1,125 target genes and 147 miRNAs were identified and considered as the miRNA binding site prediction data set. Most of the target genes have one predicted miRNA binding site (an average of 1.08 for duplicates and 1.02 for the singletons). We found that among all targets 92% are duplicates whereas 8% are singletons (fig. 1*A*). Consistent with the experimental data, this result shows that duplicates are more likely to be regulated by miRNAs than singletons in *A. thaliana* (*P* < 1e-6, chi-square test). To test whether the result might be affected by the stringent criterion used to predict miRNA targets, we did the same analysis using the three prediction methods separately. They gave similar results and reflected the same trends (*P* < 1e-7) (supplementary fig. S1, Supplementary Material online). In addition, we repeated the same analyses using duplicated genes defined with the *E*-value cutoff as less than 1e-20 and 1e-30. In both analyses, duplicates are overrepresented in both the experimental data set and the binding site prediction data set (supplementary table S1, Supplementary Material online). Overall, the results from both prediction and experimental data indicate a preferential role of miRNA regulation in duplicated genes in *A. thaliana*.

### miRNA Target Sites Have Diverged Extensively in Duplicated Genes

To assess the conservation of miRNA binding sites between duplicated genes, we analyzed all pairs of duplicates with at least one gene as an miRNA target to determine whether they have the same or divergent miRNA binding sites. We used alpha whole-genome duplicates, tandem duplicates, and other types of duplicates in the analyses (supplementary table S1, Supplementary Material online). Divergent miRNA binding site patterns were detected if only one of the two paralogous genes has an miRNA binding site, or if both of the genes have miRNA binding sites but the binding sites are different. In cases where at least one gene in a paralog pair is an miRNA target, 91% and 68% of the paralog pairs were observed to show divergent patterns of miRNA binding sites in the miRNA binding site prediction data set and experimental data set, respectively (table 1; supplementary table S3, Supplementary Material online). Among the paralog pairs with divergent patterns of miRNA binding sites, most of the pairs have only one gene as an miRNA target (95% and 93% for the miRNA binding site prediction data set and the experimental data set, respectively). Others show both duplicates with binding sites but these binding sites are by different miRNAs.

**Table 1:** Conservation and Divergence of miRNA Binding Site Patterns in Duplicated Genes in *Arabidopsis thaliana*

	WGD	TD	Others	Total
miRNA binding site prediction data set				
Same	21	8	22	51
Divergent	211	65	231	507
Total	232	73	253	558
Experimental data set				
Same	12	1	7	20
Divergent	14	9	20	43
Total	26	10	27	63

Note.—The numbers of paralog pairs showing the same or divergent miRNA binding site patterns based on the miRNA binding site prediction data set and the experimental data set are indicated. Each category (same, divergent, and total) of miRNA binding site pattern is divided into three classes corresponding to the three types of duplicated genes, from left to right, whole-genome duplicates (WGD), tandem duplicates (TD), and other types of duplicates (others).

We also determined whether there is any difference in the proportion of divergent miRNA binding site patterns among all three classes of duplicated genes. Considering the small sample size of the experimental data set, the analysis was limited to the binding site prediction data set. We found that 91%, 89% and 90% of paralogous gene pairs were shown to have divergent miRNA binding sites for whole-genome duplicates, tandem duplicates and other types of duplicates, respectively (table 1). No significant difference was detected among them (*P* > 0.1, chi-square test). Altogether, the above results indicate a large divergence of miRNA binding site patterns between duplicated genes, but different types of duplicated genes do not show differences in this regard.

### Divergence in miRNA Binding Sites in Genes Derived from whole genome triplication in *Brassica* *rapa*

To extend the study to another species and to analyze miRNA binding sites in duplicated genes derived from a more evolutionarily recent WGD event than the alpha-WGD in the Brassicaceae, we used the WGT event that occurred in the ancestor of extant *Brassica* species after the split with the *Arabidopsis* lineage at about 17–20 ma (Yang et al. 1999; Lysak et al. 2005; Parkin et al. 2005). Duplicated genes derived from the WGT have been identified (Wang et al. 2011). We used *B. rapa* for analysis because it has the largest number of currently identified miRNA genes among *Brassica* species in miRBase. Considering the limited number experimentally verified miRNA targets in *Brassica*, only the three miRNA binding site prediction methods were used. Similar to the analyses in *A. thaliana*, protein-coding genes predicted to be miRNA targets by at least two of three prediction programs were included in the prediction data set for *B. rapa*. After genome triplication, some triplicated genes retained three copies whereas others retained only one or two copies. In total, there are 70 pairs and triplets of genes derived from the WGT with at least one member predicted to be an miRNA target. Among them, 52 paralog pairs/triplets show divergence of miRNA binding sites (table 2; supplementary table S5, Supplementary Material online). Among the retained triplicates, there were more cases of two genes having an miRNA binding site than all three or just one. Thus, consistent with *A. thaliana*, the majority of duplicated genes analyzed in *B. rapa* have extensively diverged in their miRNA binding sites patterns. Moreover, the proportion of paralogous gene pairs with divergent miRNA binding sites patterns derived from the Brassica-specific WGT is significantly lower than that of *A. thaliana* for the prediction data set (*P* < 0.05, chi-square test). This could be due to the lower divergence time of paralogous genes formed by the Brassica-specific genome triplication than the alpha-WGD specific to Brassicaceae.

**Table 2:** Conservation and Divergence of miRNA Binding Site Patterns in Whole-Genome Duplicates and Triplicates in *Brassica rapa*

	Duplicates		Triplicates			Total
No. of miRNA targets	1	2	1	2	3	
Same	—	17	—	—	1	18
Divergent	34	1	0	14	3	52
Total	34	18	0	14	4	70

Note.—Numbers are indicated of paralog pairs and triplicates showing the same or divergent miRNA binding site patterns based on the miRNA binding site prediction data set for *Brassica rapa*. Genes generated through WGT are divided into duplicates and triplicates based on how many genes are retained. “No. of targets” indicates how many genes are miRNA targets (1 or 2 for duplicates and 1, 2, or 3 for triplicates).

### Duplicated Genes with Divergent miRNA Regulation Patterns Show More Divergence in Expression Patterns in *A. thaliana*

**Fig. 2.—:**
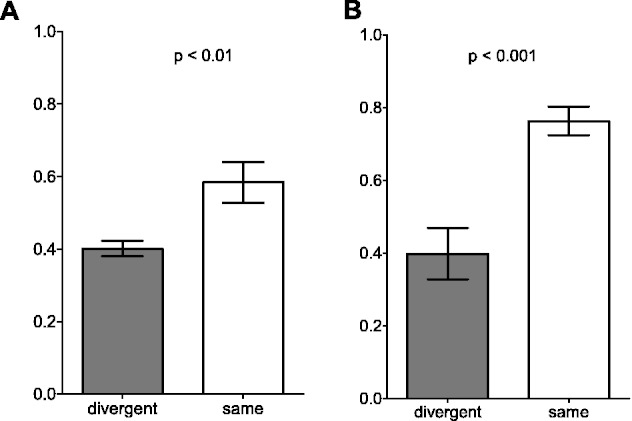
Expression correlation analysis between paralog pairs with the same and divergent miRNA regulation patterns. All paralog pairs with at least one gene targeted by an miRNA are classified into two categories based on whether they show divergent miRNA regulation patterns for both miRNA binding site prediction data set (*A*) and experimental data set (*B*). The Pearson correlation coefficient between two paralogous genes is calculated based on the microarray data with 63 different organ types and developmental stages (see Materials and Methods).

To determine whether there is a relationship between miRNA binding site divergence and expression divergence in duplicated genes, we analyzed the expression correlation between paralogous genes in *Arabidopsis* using both the binding site prediction data set and the experimental data set. (We used *Arabidopsis* and not *Brassica* for the expression analysis because much more expression data are available for *Arabidopsis*.) We used microarray data from 63 different organs and developmental stages of *A. thaliana* (see Materials and Methods). Paralog pairs with divergent miRNA binding sites show more divergence in expression patterns than those with the same miRNA target sites, indicated by their significantly lower Pearson correlation coefficient for both the target site prediction data set and experimental data set (fig. 2). Although the expression correlation coefficients vary between the two data sets, similar patterns are apparent. Thus, the divergence of miRNA binding site patterns is associated with the divergence in gene expression in *A. thaliana*.

It is possible that the group of paralog pairs with the same miRNA binding sites could show more similar expression patterns if they were formed more recently. To determine whether paralog pairs with the same binding sites are on average younger than those with divergent binding sites, we calculated Ks values for the two sets of paralog pairs. Paralog pairs with the same binding sites were detected to be younger, as a whole, than those with divergent miRNA binding sites patterns as inferred by Ks values of 1.65 for pairs with divergent binding sites and 1.16 for pairs with the same binding sites (*P* < 0.01). This suggests that younger duplicates, in general, have less divergent miRNA binding sites that could contribute to less divergence in expression patterns.

### Evolutionarily Recent miRNAs Make Major Contributions to the Divergence of miRNA Binding Patterns between Duplicates

To investigate to what extent evolutionarily recent miRNA genes contribute to the divergence of miRNA regulation of paralogous genes, we analyzed duplicated gene pairs in *A. thaliana* for targets of miRNAs that are restricted to the *Arabidopsis* genus (young miRNAs) versus those that are present in other species outside of the *Arabidopsis* genus (ancient miRNAs). We used *Arabidopsis* because of the large number of miRNAs identified in *A. thaliana* and *Arabidopsis lyrata*; in contrast, fewer miRNAs have been identified in *Brassica* species. We classified miRNAs in *Arabidopsis* as young miRNA genes or ancient miRNA genes according to whether they have homologs outside of the *Arabidopsis* genus at *E* value of 1e-10 and also based on the annotation of miRBase (see Materials and Methods). Young miRNAs in *A. thaliana* were defined as those with homologs only present in *A. thaliana* and/or *A. lyrata*. Those with homologs found outside the *Arabidopsis* genus were defined as ancient miRNAs. We analyzed the alpha whole-genome duplicates because it is known that they formed at the base of the Brassicaceae family, using miRNA targets from the binding site prediction data set.

Out of 201 duplicated gene pairs that have divergent miRNA binding sites, 104 pairs (51%) are targets of young miRNAs. In contrast, 28% (6 of 21) of paralog pairs with the same miRNA binding sites are targets of the evolutionarily young miRNAs. To see whether the results could be due to the criteria used in the identification of young miRNAs, another list of young miRNAs was generated with a BLASTN *E* value of 1e-3. No new young miRNAs were discovered and thus the results were the same. As alpha whole-genome duplicates formed at the base of the Brassicaceae family, the regulation by these young miRNAs is clearly indicative of gain of binding by miRNAs after gene duplication. This analysis demonstrates that the birth of new miRNA genes can give rise to the diversification of miRNA regulation and create differences in regulation between duplicated genes.

### Phylogenetic Analysis of Jacalin Domain Containing Proteins in *Arabidopsis* Reveals Dynamic Evolution of miRNA Targets

Based on our miRNA target predictions, we found that a family of proteins called jacalins is enriched in miRNA binding sites. Jacalins are a large family containing 56 members in *A. thaliana*. Jacalins are thought to be involved in the response to biotic or abiotic stimuli but their detailed functions are poorly understood (Yamaji et al. 2012). AT5G28520, a protein-containing jacalin domain, was found to be regulated by miR842 and miR846 (Jia and Rock 2013). In our prediction results, 18 of 49 jacalin protein sequences are predicted to be targets of at least one miRNA, with four sequences having two different miRNA binding sites. Two miRNAs, miR842 and miR846, were predicted to be miRNAs that target jacalins. Both miR842 and miR846 are only found in *A. thaliana* and *A. lyrata* indicating their recent origin after the divergence of the *Arabidopsis* genus and other species in Brassicaceae.

**Fig. 3.—:**
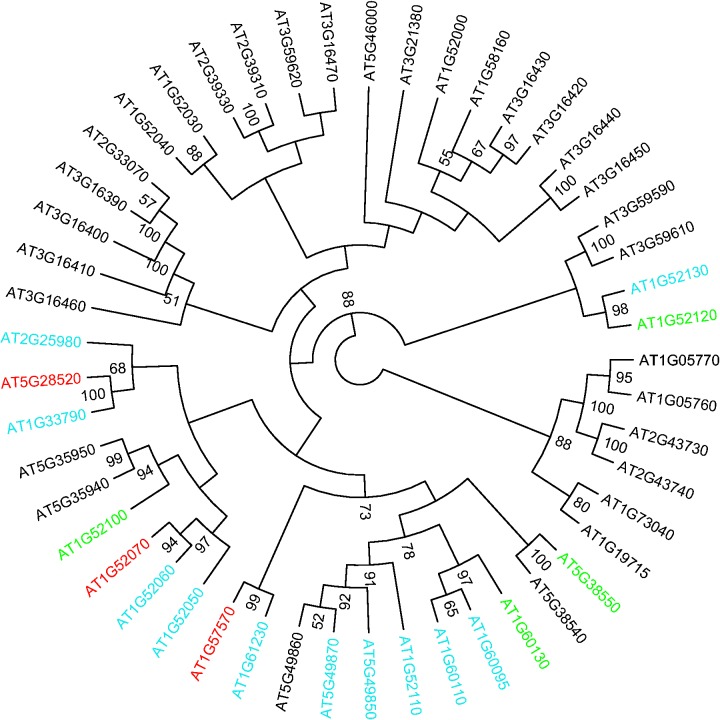
Phylogenetic analysis reveals dynamic evolution of miRNA regulation in jacalin family genes in *Arabidopsis thaliana*. Maximum-likelihood analysis was performed using RAxML. WAG+G+F+I was chosen as the most suitable substitution model based on the result of ProtTest before the phylogenetic reconstruction. Gene symbols with the color of green, blue, and red indicate targeting by miR842, miR846, and both miRNAs, respectively. Numbers next to the nodes correspond to bootstrap values obtained from 1,000 bootstrap replicates. Only the nodes with bootstrap values greater than or equal to 50 are shown in the tree.

To explore how miRNA binding sites have changed after gene duplications within the jacalin family, we reconstructed the phylogenetic history of jacalins in *Arabidopsis* and then mapped the miRNA binding sites predicted to be present in each gene on the phylogenetic tree. It appears that multiple gains and losses of miRNA binding sites events have happened during the evolution of jacalin domain containing proteins in *Arabidopsis*, although the exact number is difficult to assess. In one branch of the tree (the lower left side of fig. 3), many closely related genes potentially generated by recent duplication events show very different patterns of miRNA regulation. Some very closely related genes are targeted by different miRNAs, whereas distantly related paralogs can be regulated by the same miRNA. For example, AT5G49850, AT5G49860, and AT5G49870 were generated through TD and form one clade in the phylogenetic tree. AT5G49850 and AT5G49870 are predicted to be targeted by miR846, whereas AT5G49860 is not shown to have any miRNA binding sites possibly due to the absence of the first jacalin domain present in AT5G49850 and AT5G49870. The phylogenetic analysis of the jacalin family provides a nice example of the dynamic evolution, including multiple gains and losses, of miRNA binding sites after duplications within a gene family.

## Discussion

### Duplicates Are More Likely to be Targeted by miRNAs than Singletons

Our analyses revealed a higher fraction of duplicates as potential targets for miRNA regulation in *Arabidopsis*, indicated by both experimentally verified and predicted miRNA targets. These observations suggest an important role of miRNAs in regulating the expression of duplicated genes in *Arabidopsis*. Our study provides the first reported evidence for the preferential regulation of duplicated genes over singletons by miRNAs in plants. Our findings are consistent with a computational study in mammals (Li et al. 2008). Thus, the miRNA regulation of duplicated genes in plants and animals shows similar trends in this regard.

It has been shown that the reduction of expression levels can facilitate the retention of duplicated genes by buffering the toxic effect caused by imbalanced gene dosage (Qian et al. 2010). Hence, the enrichment of miRNA regulation in duplicated genes in *A. thaliana* suggests their contributions to maintaining gene expression balance by silencing and downregulating paralogous genes. The downregulation of expression of duplicated genes may play an important role in retention of some of them. It is possible that some genes with miRNA binding sites may avoid the negative effect caused by imbalanced dosage and be more likely to be retained after duplication. In addition, the preferential regulation of duplicates by miRNAs might be attributed to the ability of miRNA regulation to lead to tissue-specific expression divergence between paralogs. Neofunctionalization and subfunctionalization of expression patterns of duplicated genes, facilitated by miRNA regulation, could lead to retention of some duplicated genes.

### Divergence of miRNA Binding Site Patterns after Gene Duplication

After duplication genes can show divergence in expression patterns and functions. In this study, we show that a large majority of duplicated genes in *Arabidopsis* show divergent patterns of miRNA binding sites. For the data set of duplicates with experimental evidence for miRNA targeting, 68% of duplicate pairs with at least one miRNA target show clear divergence of miRNA binding sites. For the data set based on prediction results, the number increased to 87%. These results demonstrate that a large majority of duplicates show different miRNA regulation patterns no matter which data set was utilized in the analyses. We did not find a significant difference among the different types of duplicates (WGDs, tandems, other duplicates) in regards to their miRNA binding site divergence levels. Thus, the mechanism of gene duplication probably does not have an effect on the evolution of miRNA binding sites.

To extend the study to another species and examine a more recent case of polyploidy, we studied genes duplicated by the WGT in *Brassica*. Similar to duplicates in *A. thaliana*, triplicated genes in *B. rapa* have diverged extensively with respect of their miRNA binding sites. As there can be up to three paralogs derived from the Brassica-specific WGT event retained in the genome of *B. rapa*, one could hypothesize that the genes might have more divergent miRNA regulation. However, our analysis shows that the extent to which miRNA binding sites have diverged in *B. rapa* is less than in whole-genome duplicate pairs in *A. thaliana*. We think that this is possibly because the *Brassica*-specific genome triplication occurred more recently than the alpha-WGD specific to the Brassicaceae family. The shorter divergence time for triplicated genes in *B. rapa* may lead to less divergence in their miRNA regulation compared with *A. thaliana*. However, it should be noted that miRNA genes identified in *B. rapa* are likely incomplete. A more comprehensive analysis of miRNA binding site divergence after genome triplication might be performed when a more complete set of miRNA genes is available in *B. rapa* as well as other species within the *Brassica* genus.

Divergence in miRNA binding sites between duplicated genes may have an impact on their expression patterns and functions. Our observation that paralogs with divergent miRNA binding sites tend to show a greater divergence in expression profiles supports that possibility. In some cases, the divergent patterns of miRNA regulation may lead to the differential expression between paralogs. For example, in *Arabidopsis* allopolyploids, nonadditive expression of duplicated miRNAs led to expression level differences between their duplicated target genes in some cases (Ha et al. 2009).

### Evolutionarily Recent Gain of miRNA Regulation

We identified miRNAs that are specific to the *Arabidopsis* genus after the divergence of its lineage from the *Brassica* lineage within the Brassicaceae family that we refer to as young miRNAs. We present evidence that 51% of divergent miRNA regulation patterns between paralogs derived from WGD, analyzed in *A. thaliana,* can be attributed to young miRNAs that were born after the paralogs originated by duplication. Thus, it could be inferred that the divergence in miRNA binding sites between paralogs can occur by gain of miRNA regulation by the binding of a newly born miRNA. Thus, sequence changes in the coding region or UTR would not necessarily be needed for miRNA regulation to be gained. Because miRNA binding sites are often localized in coding regions in plants instead of in 3′-UTRs as in animals (Millar and Waterhouse 2005; Chen and Rajewsky 2007), it is thought that it is more difficult for genes in plants to gain regulation by an miRNA by the accumulation of point mutations (Chen and Rajewsky 2007). However, if divergent miRNA binding site patterns are caused by miRNAs born after the gene duplication occurred, point mutations would not be needed. There are several ways in which new miRNAs can arise in plants (reviewed in Nozawa et al. 2012). miRNAs could be generated through the duplication of preexisting miRNAs (Maher et al. 2006), transition of miniature inverted-repeat transposable elements (Piriyapongsa and Jordan 2008), inverted duplication of protein-coding genes (Allen et al. 2004), and spontaneous mutations in intergenic regions (De Felippes et al. 2008). The inverted duplication of protein-coding genes is of particular interest in terms of duplicated genes gaining miRNA regulation. This is because a newly born miRNA through this mechanism will have the same sequence as the protein-coding gene from which it originates (Allen et al. 2004). Therefore, the protein-coding gene from which the miRNA originates may become an miRNA target without changes in the coding sequences. Additionally, it is plausible that a new miRNA happens to have nearly perfect complementary to a sequence of a protein-coding gene through random mutations allowing for miRNA targeting. Thus, there are several ways in which new miRNAs can be created. Our results emphasize the important role of young miRNAs in regulation of duplicated genes.

## Supplementary Material

Supplementary figures S1 and S2 and tables S1–S5 are available at *Genome Biology and Evolution* online (http://www.gbe.oxfordjournals.org/).

Supplementary Data
