# Supplementary material for: Duplicate Gene Divergence by Changes in MicroRNA Binding Sites in Arabidopsis and Brassica
Source: Genome Biol Evol. 2015 Feb 2;7(3):646–55. doi: 10.1093/gbe/evv023 (PMC5322543; doi:10.1093/gbe/evv023)
Supplement: Supplementary Data [file supp_7_3_646__index.html]

Duplicate Gene Divergence by Changes in microRNA Binding Sites in Arabidopsis and Brassica — Duplicate Gene Divergence by Changes in MicroRNA Binding Sites in Arabidopsis and Brassica — Supplementary Data 

# Duplicate Gene Divergence by Changes in MicroRNA Binding Sites in *Arabidopsis* and *Brassica*

## Supplementary Data

files

**Files in this Data Supplement:**

- Supplementary Data - pdf file
- Supplementary Data - docx file
- Supplementary Data - xlsx file
- Supplementary Data - xlsx file
- Supplementary Data - xlsx file
- Supplementary Data - xlsx file
- Supplementary Data - xlsx file
