# Supplementary material for: Duplicate Gene Divergence by Changes in MicroRNA Binding Sites in Arabidopsis and Brassica
Source: Genome Biol Evol. 2015 Feb 2;7(3):646–55. doi: 10.1093/gbe/evv023 (PMC5322543; doi:10.1093/gbe/evv023)
Supplement: Supplementary Data [file supp_evv023_Supplemental_figures.pdf]

A

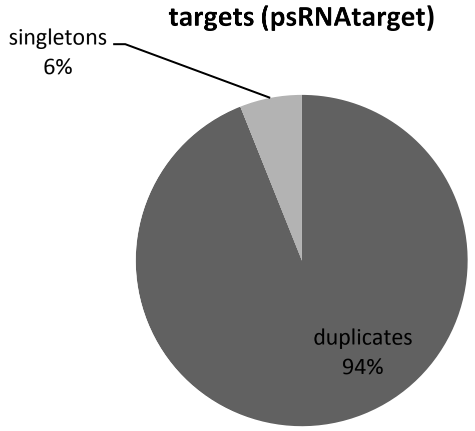

targets (psRNAtarget)
singletons
6%
duplicates
94%

B

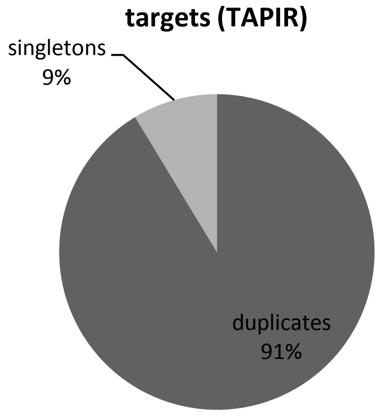

targets (TAPIR)
singletons
9%
duplicates
91%

C

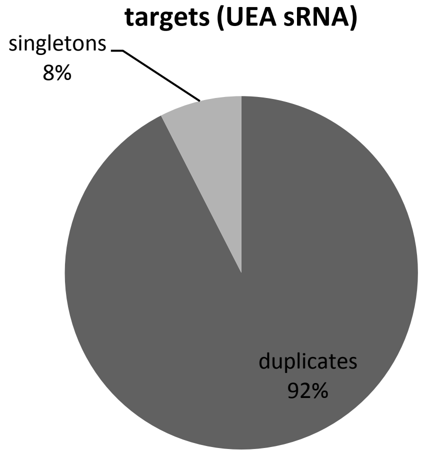

targets (UEA sRNA)
singletons
8%
duplicates
92%

```
                  10        20        30        40        50        60        70        80        90
          ....|....|....|....|....|....|....|....|....|....|....|....|....|....|....|....|....|....|
AT3G59590 ------------------------------------------------------------------------------------------
AT3G59610 ------------------------------------------------------------------------------------------
AT1G19715 ------------------------------------------------------------------------------------------
AT3G59620 ------------------------------------------------------------------------------------------
AT1G73040 ------------------------------------------------------------------------------------------
AT5G46000 ------------------------------------------------------------------------------------------
AT2G33070 ------------------------------------------------------------------------------------------
AT3G16390 ------------------------------------------------------------------------------------------
AT3G16410 ------------------------------------------------------------------------------------------
AT3G16400 ------------------------------------------------------------------------------------------
AT1G52000 ------------------------------------------------------------------------------------------
AT2G39330 ------------------------------------------------------------------------------------------
AT2G39310 ------------------------------------------------------------------------------------------
AT3G16470 ------------------------------------------------------------------------------------------
AT5G38540 ------------------------------------------------------------------------------------------
AT5G38550 ----------------------------------MIQKLGAKGIKSDERNQREWDDGSEHDDVTKIYVRGGREGIRSIYFNYVKNGKPKD
AT1G52120 ------------------------------------------------------------------------------------------
AT1G52130 ------------------------------------------------------------------------------------------
AT1G52110 MYNYSRSPGFYAKKILKMSIKRMKKKNEMSSVVDLIKIGRNCPSAIVQSDVSLSSTIIGQRFLSKIYVQYSPKGIQSIRFSYINSGKPID
AT5G49850 --------------------------------------MTERSEALGKDGNRRWDDKSDHDDVTKIYVNYSLMGIESIRFDYVKSGKPIE
AT5G49870 ------------------------------------MTERLEAEGSRRGNNSKWDDKSDHDDVTKIYVNYSLSGIESIRFDYVKSGKPKD
AT5G49860 ------------------------------------------------------------------------------------------
AT1G60130 -------------------------MSQVFSNLFQMTQRLEAQGQKTLDSPFVWDDGSDHDDVTKIHVRGDSRGINCIRFDYIKSGQRKY
AT1G60095 ------------------------------------------------------------------------------------------
AT1G60110 -----------------------------------MAQRLEAEGNKNFKGKSKWDDGSDKDDIGKISVRCEDGGITYIRFDYIKSGQPQY
AT3G16460 --------MSWDDGSHAKVKKVQLTFDEIIYSIQVTYDGATALQSQLRGSVGPKSAEFTLAPDEYITALSAYGKSLSTQEVITALTFTTN
AT1G52030 ---------------------------------------MSEKVGAMGGNKGGAFDDGVFDGVKKVIVGKDFNNVTYIKVEYEKDGKFEI
AT1G52040 ------------------------------------------------------------------------------------------
AT3G21380 ------------------------------------------------------------------------------------------
AT3G16430 ------------------------------------------------------------------------------------------
AT3G16420 ------------------------------------------------------------------------------------------
AT3G16440 ------------------------------------------------------------------------------------------
AT3G16450 ------------------------------------------------------------------------------------------
AT1G52100 ------------------------------------------------------------------------------------------
AT5G35950 ------------------------------------------------------------------------------------------
AT5G35940 ------------------------------------------------------------------------------------------
AT1G58160 ------------------------------------------------------------------------------------------
AT1G57570 -------------MERNLLSILMRRRLAERNRDAIELAAVQKMEVIGSTEGYTFDDGSDHDDVTKIFVGGGRQGIHYIEFEYVKNGQLES
AT1G61230 ------------------------------------------------------------------------------------------
AT1G52050 ------------------------------------------------------------------------------------------
AT1G52060 ------------------------------------------------------------------------------------------
AT1G52070 ------------------------------------------------------------------------------------------
AT2G25980 ------------------------------------------------------------------------------------------
AT5G28520 ------------------------------------------------------------------------------------------
AT1G33790 --------------------------------------MAQKLEAKGGKGGNQWDDLLDHDNIAKIHVQGGHEGIQYVKFDYVKFDNLKI
AT1G05760 ------------------------------------------------------------------------------------------
AT1G05770 ------------------------------------------------------------------------------------------
AT2G43730 ------------------------------------------------------------------------------------------
AT2G43740 ------------------------------------------------------------------------------------------
```

```
              100       110       120       130       140       150       160       170       180
          ....|....|....|....|....|....|....|....|....|....|....|....|....|....|....|....|....|....|
AT3G59590 ------------------------------------------------------------------------------------------
AT3G59610 --------------------------------------------------------------------------------------MMQP
AT1G19715 ------------------------------------------------------------------------------------------
AT3G59620 ------------------------------------------------------------------------------------------
AT1G73040 ------------------------------------------------------------------------------------------
AT5G46000 ------------------------------------------------------------------------------------------
AT2G33070 ------------------------------------------------------------------------------------------
AT3G16390 ------------------------------------------------------------------------------------------
AT3G16410 ------------------------------------------------------------------------------------------
AT3G16400 ------------------------------------------------------------------------------------------
AT1G52000 ------------------------------------------------------------------------------------------
AT2G39330 ------------------------------------------------------------------------------------------
AT2G39310 ------------------------------------------------------------------------------------------
AT3G16470 ------------------------------------------------------------------------------------------
AT5G38540 MLIYNADSVVFLFSLFLQFEINHLQDEHLESVDAYYNKKSYGLQAIQFKTNFRTSELMGYS----YECTMFTLAVKGKKIIGFHGSDNVH
AT5G38550 GSIHGYFDSGFTQTFEINHLR----GEYLESVDAYYDKKSYGMQAIQFKTNFRTSELMGYS----YECTMFTLAVQGKKIIGFHGSNYVH
AT1G52120 ------------------------------------------------------------------------------------------
AT1G52130 ------------------------------------------------------------------------------------------
AT1G52110 GSLHGQSDNTYTQTFEINHLK----HEHLESVEGYYTDDTG-IQALQFKTNLRISEPMGYH---D-GCTKFILAIEGKKIIGFHGLSFEG
AT5G49850 GPFRGETYNTYTHTFEINHLK----NEHLESVEGSYTQR--GIQTLQFKTNLRISEPIGYP---GKDGIKFILAVEGKKIIGFHGSTYFR
AT5G49870 GPFRGQSYNTYTHTFEINHLK----YEHLESVEGYYTEDT-GIEALQFKTNLRISEPIGYH---HDGCTKFILAVEGKKIIGFHGSSFAL
AT5G49860 ------------------------------------------------------------------------------------------
AT1G60130 KSFYGPSWAGFTQTFKINHKE----DEQLESVEGFYKPDSRTIVGLQFKTNLRISELIGHG---KKDDTKFSLAVDGKKIIGFHGCSGSY
AT1G60095 -------------MDRSMFEIDHKKDEQLVSVEGYCKPGSDLIQGVQFKTNLRISEIIGFEKAIFGNPTTFSLAEDGKKIIGFHGYSMAN
AT1G60110 NTFPGNPGRGILQTFDINHKN----DEHLESVEGYYDPKSDAIKGLQFKTNMRISELIGYA---NDGATKFSLAVEGKKIIGFHGAYNTY
AT3G16460 KTSYGPYGTKSGFQISAPEATGKQISGFLGTSGNVLNTIDVHYSPIPTGGTGTGGTGTGTGTGTGTGTGTGTGTGTGTGTGTGGTGTG
AT1G52030 REHGTNRGQLKEFSVDYPNEYITAVGGSYDTVFGYGSALIKSLLFKTSYGRTSPILGHTTLLGNPAGKEFMLESKYGGKLLGFHGRSGEA
AT1G52040 ------------------------------------------------------------------------------------------
AT3G21380 ------------------------------------------------------------------------------------------
AT3G16430 ------------------------------------------------------------------------------------------
AT3G16420 ------------------------------------------------------------------------------------------
AT3G16440 ------------------------------------------------------------------------------------------
AT3G16450 ------------------------------------------------------------------------------------------
AT1G52100 ------------------------------------------------------------------------------------------
AT5G35950 ------------------------------------------------------------------------------------------
AT5G35940 ------------------------------------------------------------------------------------------
AT1G58160 ------------------------------------------------------------------------------------------
AT1G57570 GVHLGVRYRGFTETFEINHLN----NEHLESVEGYYDYGSGYIQGLQFKTNFRVSELIGYD-----EGTKFSLSVKGKRIIGFHGYMKER
AT1G61230 ----------------MQIEIDHLNNEHLESVESYYDDAPCYIQALQFKTNFKVSELIGYG-----KGTKFSLSVKGKIIIGFHGYIKSQ
AT1G52050 ------------------------------------------------------------------------------------MFIIYL
AT1G52060 ------------------------------------------------------------------------------------MIFIYI
AT1G52070 ------------------------------------------------------------------------------------MVIIYI
AT2G25980 ------------------------------------------------------------------------------------------
AT5G28520 ------------------------------------------------------------------------------------------
AT1G33790 GQPKLGSIHGLSRKGFTQTFEIDPTSEYIVSVEGYYDESKGIIQALKFKTNKKTSDMIGYD----ENGLKFSLEVKGKAIIGFHGFADTN
AT1G05760 ------------------------------------------------------------------------------------------
AT1G05770 ------------------------------------------------------------------------------------------
AT2G43730 ------------------------------------------------------------------------------------------
AT2G43740 ------------------------------------------------------------------------------------------
```

```
                   190       200       210       220       230       240       250       260       270
              ....|....|....|....|....|....|....|....|....|....|....|....|....|....|....|....|....|....|
AT3G59590    ------------------------------------------------------------------------------------------
AT3G59610    DHDLPYDLEGEILSHLPI---------------------------------------------------------------QILARFRC-
AT1G19715    ----------MSVEGKPA---------------------------------------------------------------SLGPWGGQ-
AT3G59620    ------------------------------------------------------------------------------------------
AT1G73040    ------------------------------------------------------------------------------------------
AT5G46000    --------------MSKK---------------------------------------------------------------VTIAYGGS-
AT2G33070    ---------------MVQ---------------------------------------------------------------KVEARGGE-
AT3G16390    ---------------MAQ---------------------------------------------------------------KLVAQGGE-
AT3G16410    ---------------MAQ---------------------------------------------------------------KVEAQGGN-
AT3G16400    ---------------MAQ---------------------------------------------------------------KLEAKGGE-
AT1G52000    ----------------MS------------------------------------------------------------------------
AT2G39330    ------------MAKIYR---------------------------------------------------------------KLTGYGGE-
AT2G39310    ------------MAKMYR---------------------------------------------------------------KLALCGGE-
AT3G16470    ---------------MAK---------------------------------------------------------------KLEAQGGR-
AT5G38540    ----IYSLGAYFTSITPT---------------------------------------------------------------RLEVKGGM-
AT5G38550    ----ILSLGAYFISIAPT---------------------------------------------------------------RLEVKGSK-
AT1G52120    ---------MSQDSNALE---------------------------------------------------------------MIQNEGPK-
AT1G52130    ---------------MTQ---------------------------------------------------------------KLESVGSER
AT1G52110    RR--VYSLGAYFTWITPT---------------------------------------------------------------RIEAIGGK-
AT5G49850    ----LYSLGAYFTRVTPT---------------------------------------------------------------RIEAIGGK-
AT5G49870    R---VGSLGAYFTWDTPT---------------------------------------------------------------IIKAVGGK-
AT5G49860    ------------------------------------------------------------------------------------------
AT1G60130    ----LESLGAYFTCIAPT---------------------------------------------------------------RMEAKGAK-
AT1G60095    ----LNSLGAYFTWISPT---------------------------------------------------------------KMEAKGGK-
AT1G60110    ----LNSLGAYVTWIVPT---------------------------------------------------------------KLKAKGGK-
AT3G16460    TGTGTGGTGTGTGTGGTGTGTGTGTGTGTGTGTGTGTGGTGTGTGTGGTGTGTGTGTGTGTGGTGTGTGTGTGSGAQKLEAQGNST
AT1G52030    ----LDAIGPHFFAVNSS-------------------------------------------------------------LKHFKPQGGN-
AT1G52040    -----------MSTGGPQ---------------------------------------------------------------KLEAQGGK-
AT3G21380    ---------------MAA------------------------------------------------------------------------
AT3G16430    ------------------------------------------------------------------------------------------
AT3G16420    ------------------------------------------------------------------------------------------
AT3G16440    ------------------------------------------------------------------------------------------
AT3G16450    ------------------------------------------------------------------------------------------
AT1G52100    ---------------MAL---------------------------------------------------------------KVEAKGGK-
AT5G35950    ---------------MAL---------------------------------------------------------------MVKAEGGN-
AT5G35940    ---------------MAL---------------------------------------------------------------MVNAKGGN-
AT1G58160    ------------------------------------------------------------------------------------------
AT1G57570    K---IISLGGYFSWIHPR---------------------------------------------------------------KMEAKGSK-
AT1G61230    NNPLVKSLGAYFTWIPDC---------------------------------------------------------------RLEAKGSK-
AT1G52050    FIFLSSAIIDSNGVAMAQ---------------------------------------------------------------KIEAIGGK-
AT1G52060    FLFLSSAIIDSNGFAMAQ---------------------------------------------------------------KLEAKGGK-
AT1G52070    FLFLSSAIIDSTGLAKAQ---------------------------------------------------------------KLDAIGGK-
AT2G25980    ---------------MAQ---------------------------------------------------------------RLEAKGGK-
AT5G28520    ---------------MAQ---------------------------------------------------------------KLEAIG---
AT1G33790    ----LNSLGAYFAPAPPTKFDYQGRTSPIFGKVAARKFVFESNGSALIGFHGRAAAAVDAIGAYFSPLILSAPAPPPPPAEKLQAKGGN-
AT1G05760    ------------------------------------------------------------------------------------------
AT1G05770    ------------------------------------------------------------------------------------------
AT2G43730    ------------------------------------------------------------------------------------------
AT2G43740    ---------------MKS------------------------------------------------------------------------
```

```
                280       290       300       310       320       330       340       350       360
             ....|....|....|....|....|....|....|....|....|....|....|....|....|....|....|....|....|....|
AT3G59590   ------MELSF---KREKICSVEVNLDVPSIE--------VHSHNLPSYIPGYNGF----------FMTVEYCDGLVVYATENGIGICNP
AT3G59610   VCKR--WNTLF---KERRFFNSDLGLARPQFILLAESKICSVDVNLDGPSIEVHNLPSDIPGYKLYMPM--HVEYCDGLFLYATCYGIGI
AT1G19715   SGHA--WDDGM----YTTVKQIIIAHG-SGIDSIQIEYD-KNGSSVWSEKRGGKGGKKF-DKVKFDYPH-EYLISVNGTYGS-FDVWGTI
AT3G59620   ------------------------------------------------------------------------------------------
AT1G73040   ------MDQ---------------------------------------------------------------------------------
AT5G46000   NGNK--FDDGVYEGTYDGVRKLIVGEDFHGIVYLKIQYV-KNGDVVVKEHGRARGTHITETEFEVKCPD-EYITSIWGTCRN--------
AT2G33070   IGDV--WDDGA----YDGVRKVYVGQGEDGIAFVKFEYV-NGSQEVVGDERGKKTLLG-AEEFEVDPDD--YIVYVEGYHEKVFGVTTKE
AT3G16390   TGDV--WDDGV----YDNVTKVYVGQGQYGIAFVKFEYA-NGSEVVVGDEHGEKTELG-VEEFEIDSDD--YIVYVEGYREKVSDMTS-E
AT3G16410   GGNQ--WDDGS---EYDAVTKIQVAAGGNGIEYVKFTYV-KNGQTEEAPLRGVKGRSFEADPFVINHPE-EHLVSVEGRYNP------EG
AT3G16400   MGDV--WDDGV----YENVRKVYVGQAQYGIAFVKFEYV-NGSQVVVGDEHGKKTELG-VEEFEIDADD--YIVYVEGYREKVNDMTS-E
AT1G52000   ------WDDGK----HTKVKRVQLTFD-DVIRSIEVEY---DGTSLKSQPRGTAGT----KIDGFTLSSDEYITEVNGYYKT---TFSGE
AT2G39330   GGRE--WDDDV----YEGVRKVYVGQDINRITYVKFEYVKEDGQVVTTEYGKIIQQ---PKEFVLQYPD-EHIIAVEGNYRG-VALCATE
AT2G39310   GGQE--WDDDV----YEGVRKVYVGQDLNRITYIKFEYVQEDGEVVTTEYGTTNQH---PKEFVIQYPD-EHIIAVEGSYHQ-VALIATE
AT3G16470   GGEE--WDDGG---AYENVKKVYVGQGDSGVVYVKFDYE-KDGK-IVSHEHGKQTLLG-TEEFVVDPED--YITSVKIYYEK-LFGSPIE
AT5G38540   GGKK--WEDGF---DHDNVSKIQVLGGFEGILYIKVDYI-KNGKLETGLIHGDSGGDGFLQKMEINQSKNEYLVYVEGYYDD-----ASE
AT5G38550   GSKK--WDDGF---DHENVSKIEVLGGFEGILYIKVDYI-KNGKLETGLVHGHSGGDGFLQKMEINQSKNEYLVYVEGYYDD-----ASE
AT1G52120   ------WDDGF---DHDDVTKIYLVGGKTGIDFIKIDYV-KSGKPKNGPFHGYSGG-GFLQMFEIDNLKNEYLESVEGYYTN----RSGE
AT1G52130   KSSEYMWDDGS---EHDDVTKIYVRGGTKGIEFIKFGYV-KAGELLDGSFHGYSDT-GFTQMFEIDHRKNEHLLSVEGYFDY-----YND
AT1G52110   VGTK--WDDGV---DHAGFTKIHVRSGPKGIQYIKFLYVDKYGHLKDGPIHGSISG------------------KVEGYYND---HDESG
AT5G49850   VGTK--WDDGV---DQAGFTKIHVRSGQEGIQFIKFEYVDKNGRLRDGSIHGSIYRRGSPHVFEIRHVDKEYLVSVEGYYDG---DGDCA
AT5G49870   VGTK--WDDGV---NQAGFTKIHVRSGQKGIQFIKLEYVDKDGNLTDGPIHGSIYRRGSPHVFEFKHDE-EYLVSVEGYYEG---DEECE
AT5G49860   ------------------------------------------------------------------------------------------
AT1G60130   GGTD--WNDGA---DHEGVAKIYVRGGRDCIQYIKFDYV-KDRKYIYGPAHGVRGR-GFTESFEINHLDNEYMVSVEGYYDE----GDSG
AT1G60095   GGTE--WNDGA---EHEGFTKIYVQGGCDGIQYIKFDYV-KDGQHKYGSPHGVKGS-ESTEPFEINHLDKEYLISVEGYYDE----GDSG
AT1G60110   GGKE--WNDGA---DHEGITKIYVRGGYEGLQYVKFDYI-KDGQQIYGSPHGVRGR-GFTELFEINHLDKEYLISVEGYYDE----GESG
AT3G16460   GGTS--WDDGS---DYDGVTKIYASYGGEGIQYVKFDYV-KGGVTKQGVLHGKQQSRQNPREFVINHPD-EYLVSVEGWYET--------
AT1G52030   GGSA--WDDGA----FDGVRKVLVGRNGKFVSYVRFEYA--KGERMVPHAHGKRQE--APQEFVVDYPN-EHITSVEGTID--------G
AT1G52040   EGKE--WDDGA---EHDGVTKIYVAAGGLGIEQIRFDYV-KNGQPKEGSFHGVKGR-STISTIEISHPA-EYLISMEGWYDS------TN
AT3G21380   ATMS--WDDGK----HMKVKRVQITYE-DVINSIEAEY---DGDTHNPHHHGTPGK----KSDGVSLSPDEYITDVTGYYKT---TGAED
AT3G16430   ------------------------------------------------------------------------------------------
AT3G16420   ------------------------------------------------------------------------------------------
AT3G16440   ------------------------------------------------------------------------------------------
AT3G16450   ------------------------------------------------------------------------------------------
AT1G52100   VGIQ--WDDGS---DYHDVTKIYVRGGLEGIQFIKFEYV-KAGKKVIGPIHGASGR-GFTETFEINNLEKEYLLSIEGYYNA-----STG
AT5G35950   GGKR--WDDGF---DYEGVTKIYVRGGLEGIQFIKFDYV-KDGKTITGPIHGVSGR-GLTQTFEINHLQKEYLLSIEGYYDI-----STG
AT5G35940   GGKE--WDDGF---DYEGVTKIHVRAGSEGIQFIKFDYV-KVGKTIDGPIHGVSGL-GMTQTFEINHLQKEYLVSIEGYYDK-----STG
AT1G58160   ------------------------------------------------------------------------------------------
AT1G57570   GGNQ--WDDGT---NNDGVTKIHVRGGVEGIQYIKFDYVRKSGQHINGSIHGLSGS-GFTQTFEIDHLNNEHLVCVEGYYDD-----ESG
AT1G61230   GGIQ--WDDGA---DHEGITKIHVRGGFEGIQYIKFDYV-KSGQPKIGSVHGLS----------------------EGYYDD-----EST
AT1G52050   GGKR--WDDGA----NDNVAKVYIRGDHEGIQYIKFDYV-KDGQSFNGSVHGVSAD-GFTQTFEIDHLQYEQIVSVEGYYDW-----KTG
AT1G52060   GGKE--WDDGA---GHDNVAKVYIRGGLEGIQYIKFDYV-KDGQSVEGSIHGVSGS-GFTQMFEIDYQNGEHIVSVDGYFDK------SG
AT1G52070   GGKQ--WDDGA---DHDNVAKVYIRGGLEGIQYIKFDYV-KDGKTIDASIHGVSGS-GFTQTFEIDYQNSEYIVSVDGYYDK------SG
AT2G25980   GGNQ--WDDGA---DHENVTKIHVRGGLEGIQFIKFEYV-KAGQTVVGPIHGVSGK-GFTQTFEINHLNGEHVVSVKGCYDN-----ISG
AT5G28520   RGLQ--WDDSS---DHDNVTKILVRGGREGIQYVKFDYV-KSGQPQTGLIHGLSGRGGFTQTFEIDQKD-EHLVSVEGYYDV-----TKG
AT1G33790   GGNQ--WDDLA---DHDHVTKIYVQGGQEGIQYVKFDYV-KNGQPQSGSVHGLLGR-GFTQTFEIDPTN-EHLVSVEGYYDE-----SKG
AT1G05760   ------------------------------------------------------------------------------------------
AT1G05770   ----------------------------------------MEGKIKIGP-----------------------------------------
AT2G43730   ------------------------------------------------------------------------------------------
AT2G43740   ------------------------------------------------------------------------------------------
```

```
                370       380       390       400       410       420       430       440       450
            ....|....|....|....|....|....|....|....|....|....|....|....|....|....|....|....|....|....|
AT3G59590   LLRQIRWIKSKVNYRYNGVGYDNSR----------PENHYKIFES-CPYSDTTVKASITEFVSDAWIS----------------------
AT3G59610   CNPWLRQ-IRWFKSSYEGYDFSG-------------------MGYDNSRQDKHYKILGSYCTN---------------------------
AT1G19715   CVRSLTF-ESNR-RKYGPFGVDS-----GTFFAL-PKSGSKIIGF-HGKAGWYLDAIGVHTQPIPKENNPSSKILLHSHQSFSQGDKKHE
AT3G59620   -----------------------------MAFRM--------------------------------------------------------
AT1G73040   ----------------------------------------------QQQGDKNLT-----------------------------------
AT5G46000   --DHHRY-KSTGGRTFEQL--TAAD---KYDINL-AAKMGTPMYW-NTVSELQFKTSHGRTS----------------------------
AT2G33070   IISTLTF-KTYKGKTSPPFGIVS-G----TKFVL---QGGKIVGF-HGRSTDVLHSLGAYIS----------------------------
AT3G16390   MITFLSF-KTSKGKTSQPIVKKP-----GVKFVL---HGGKIVGF-HGRSTDVLHSLGAYVS----------------------------
AT3G16410   LILGLTF-KSNK-KTSDLIGYED-----GTPFTL-QVQDKKIVGF-YGFAGNNLHSLGAYF-----------------------------
AT3G16400   MITFLSI-KTFKGKTSHPIEKRP-----GVKFVL---HGGKIVGF-HGRSTDVLHSLGAYVS----------------------------
AT1G52000   VITSLTF-KTNK-RTYGTYGNKT-----SSYFSVAAPKDNQIVGF-LGSSSHALNSIDAHFAPAPPPGSTGAKPGASGIGSDSGSIGSAG
AT2G39330   VITNLVF-KTSKGRKSPLFGPNLLGITTGTKFVI-EDGGKKIVGF-HGRSGNALDALGVYFVHGSLTTS---------------------
AT2G39310   VITSLVF-KTSK-------------------------------GF-HGRAGDAVDALGVYFVLDT-------------------------
AT3G16470   IVTALIF-KTFKGKTSQPFGLTS-----GEEAEL---GGGKIVGF-HGSSSDLIHSVGVYIIPST-------------------------
AT5G38540   TIQGLHF-QTNL-NNPVMMGYKK-----GRKFLL-ASNGNKIIGF-HGYADKSLNSLGAYFSRA--------------------------
AT5G38550   TIQGLHF-QTNL-NNPVMMGYKK-----GRKFLL-ASNGNKIIGF-HGYADKSLNSLGAYFSTT--------------------------
AT1G52120   FIGAIQF-KTNL-RVSEIIGYSYWG---LKKFKL-AKHGNKIIGF-QGSAEYRLKDLDAYFTPI--------------------------
AT1G52130   IMYAIQF-KTNL-KISEIMGYEYSG----HKFTL-AMEGKKIIGF-HGFADVNLRALGAYVTWI--------------------------
AT1G52110   VIQALRF-KTNI-KTSELMGSNT-G----KKFRL-AASEMKIVGF-HGYAGKNLRSLGAYFTPI--------------------------
AT5G49850   VIQALRF-RTNV-KTSQLMGPKT-----GKKFRL-AASGMKIVGF-HGYAEKNLTSLGGYFTPI--------------------------
AT5G49870   VIQGLQF-RTNI-KTSELMGSNT-----GKKFKL-TASGMKIVGF-HGYAEKNLSSLGAYLTPL--------------------------
AT5G49860   ------------------------------------------------------------------------------------------
AT1G60130   IIQGIQF-RTNI-KTSELIGYNN-----GKKFSL-AANGKKIIGF-HGYADQNLNSLGAYFT----------------------------
AT1G60095   VIQGIQF-KTNI-KTSELIGDKK-----GRKFSL-AANGKKIIGF-HGYADKNLNSLGAYFTT---------------------------
AT1G60110   VIQGIQF-KTNI-RTSELMGDNR-----GRKFSL-AANGKKIIGF-HGYAEKNLNSLGAYFTT---------------------------
AT3G16460   VMLGIQF-KTNL-NTYEVSIYPF-EPSTDTKFTL-QVQDKKIIGF-HGFAGNHVNSIGAYFVPKSSTTPVPS------------------
AT1G52030   YLSSLKF-TTSKGRTSPVF-----GNVVGSKFVF-EETSFKLVGF-CGRSGEAIDALGAHFAP---------LPAPTPAPAPAPAPAPAP
AT1G52040   IIQGIQF-KSNK-HTSQYFGYEFLG--DGTQFSL-QVNDNKIISF-HGFADSHLNSVGAYFAPISSSLTT--------------------
AT3G21380   AIAALAF-KTNK-TEYGPYGNKT-----RNQFSIHAPKDNQIAGF-QGISSNVLNSIDVHFAPLPS------------------------
AT3G16430   ------------------------------------------------------------------------------------------
AT3G16420   ------------------------------------------------------------------------------------------
AT3G16440   ------------------------------------------------------------------------------------------
AT3G16450   ------------------------------------------------------------------------------------------
AT1G52100   VIQCLQF-ITNK-KTYDPIGYNE-----GARFTL-SASRSKIIGF-HGFADKYLNSLGAYFIKI--------------------------
AT5G35950   VIQSIQF-KTNQ-QTSDMMGFNE-----GTKFSL-RSMRGRIIGF-HGFADKNLYSLRAYYIRI--------------------------
AT5G35940   VIQSIQF-KTNV-KTSDMMGFNK-----GTKFSL-GIIRNKIIGF-HGFSDKNVYSLGAYFIKV--------------------------
AT1G58160   ------------------------------------------------------------------------------------------
AT1G57570   VIQALQF-KTNI-KTSELLGYKK-----GKKFSL-VDKRKKIVGF-HGYADKNLNSLGAYFTTV--------------------------
AT1G61230   AIQALQF-KTNI-KTSELLGYEK-----GKKFSL-ADKGKKIIGF-HGYAEKNLISLGAYFTTV--------------------------
AT1G52050   VMQALQF-KTNL-KTSEFIGYQK-----GTKFSL-GVDGKVIVGF-HGSAWRSLRSLGAYVKT---------------------------
AT1G52060   VMQALEF-KTNR-KTSEVIGYPKSN----TKFSLGGVNGKMINGF-HGSAGKALNSIGAYLTKV--------------------------
AT1G52070   TMQALEF-KTNL-KTSEVIGYPK----GTTKFSLGGVNGKMVIGF-HGSAGKVLNSIGAYLTTA--------------------------
AT2G25980   VIQALQF-ETNQ-RSSEVMGYDDTG----TKFTL-EISGNKITGF-HGSADANLKSLGAYFTPP--------------------------
AT5G28520   VIQALKF-KTNK-KTSEMIGYDDTG----IKLSL-EVKGKKIIGF-HGYAETNLNSLGAYFTTT--------------------------
AT1G33790   LVQGLKF-KTNK-KTSDMIGYDENG----LKFSL-EVNGKKIIGF-HGYAQTYLNSLGAYFVTA--------------------------
AT1G05760   ---------------------------------------------------MKIGPVG--------------------------------
AT1G05770   ------------------------------------------------------------------------------------------
AT2G43730   ----------MKSRNREMF---------------------------------KVGPIG--------------------------------
AT2G43740   ----------------------------------------------RGREMFKVGPIGS-------------------------------
```

```
                  460        470        480        490        500        510        520        530        540
              ....|....|....|....|....|....|....|....|....|....|....|....|....|....|....|....|....|....|
AT3G59590     ------------------------------------------------------------------------------------------
AT3G59610     ------------------------------------------------------------------------------------------
AT1G19715     YSVLQGSVGQNFDIVVTLRKKDPTLPSFESRDSAGAEV----------------------------------------------------
AT3G59620     ------------------------------------------------------------------------------------------
AT1G73040     ------------------------------------------------------------------------------------------
AT5G46000     ------------------------------------------------------------------------------------------
AT2G33070     ------------------------------------------------------------------------------------------
AT3G16390     ------------------------------------------------------------------------------------------
AT3G16410     ------------------------------------------------------------------------------------------
AT3G16400     ------------------------------------------------------------------------------------------
AT1G52000     TNPGADGTRETEKNAGGSKPSSGSAGTNPGASAVGNGETEKNAGGSKPSSGSAGTNPGASAGGNGETEKNVGGSKPSSGKAGTNPGANAG
AT2G39330     ------------------------------------------------------------------------------------------
AT2G39310     ------------------------------------------------------------------------------------------
AT3G16470     ------------------------------------------------------------------------------------------
AT5G38540     ------------------------------------------------------------------------------------------
AT5G38550     ------------------------------------------------------------------------------------------
AT1G52120     ------------------------------------------------------------------------------------------
AT1G52130     ------------------------------------------------------------------------------------------
AT1G52110     ------------------------------------------------------------------------------------------
AT5G49850     ------------------------------------------------------------------------------------------
AT5G49870     ------------------------------------------------------------------------------------------
AT5G49860     ------------------------------------------------------------------------------------------
AT1G60130     ------------------------------------------------------------------------------------------
AT1G60095     ------------------------------------------------------------------------------------------
AT1G60110     ------------------------------------------------------------------------------------------
AT3G16460     ------------------------------------------------------------------------------------------
AT1G52030     APSPAPASAPVPAPAPTPAP----------------------------------------------------------------------
AT1G52040     ------------------------------------------------------------------------------------------
AT3G21380     ------------------------------------------------------------------------------------------
AT3G16430     ------------------------------------------------------------------------------------------
AT3G16420     ------------------------------------------------------------------------------------------
AT3G16440     ------------------------------------------------------------------------------------------
AT3G16450     ------------------------------------------------------------------------------------------
AT1G52100     ------------------------------------------------------------------------------------------
AT5G35950     ------------------------------------------------------------------------------------------
AT5G35940     ------------------------------------------------------------------------------------------
AT1G58160     ------------------------------------------------------------------------------------------
AT1G57570     ------------------------------------------------------------------------------------------
AT1G61230     ------------------------------------------------------------------------------------------
AT1G52050     ------------------------------------------------------------------------------------------
AT1G52060     ------------------------------------------------------------------------------------------
AT1G52070     ------------------------------------------------------------------------------------------
AT2G25980     ------------------------------------------------------------------------------------------
AT5G28520     ------------------------------------------------------------------------------------------
AT1G33790     ------------------------------------------------------------------------------------------
AT1G05760     ------------------------------------------------------------------------------------------
AT1G05770     ------------------------------------------------------------------------------------------
AT2G43730     ------------------------------------------------------------------------------------------
AT2G43740     ------------------------------------------------------------------------------------------
```

```
                    550       560       570       580       590       600       610       620       630
                ....|....|....|....|....|....|....|....|....|....|....|....|....|....|....|....|....|....|
AT3G59590       ------------------------------------------------------------------------------------------
AT3G59610       ------------------------------------------------------------------------------------------
AT1G19715       ------------------------------------------------------------------------------------------
AT3G59620       ------------------------------------------------------------------------------------------
AT1G73040       ------------------------------------------------------------------------------------------
AT5G46000       ------------------------------------------------------------------------------------------
AT2G33070       ------------------------------------------------------------------------------------------
AT3G16390       ------------------------------------------------------------------------------------------
AT3G16410       ------------------------------------------------------------------------------------------
AT3G16400       ------------------------------------------------------------------------------------------
AT1G52000       GNGGTEKNAGGSKSSSGSARTNPGASAGGNGETVSNIGDTESNAGGSKSNDGANNGASGIESNAGSTGTNFGAGGTGGIGDTESDAGGSK
AT2G39330       ------------------------------------------------------------------------------------------
AT2G39310       ------------------------------------------------------------------------------------------
AT3G16470       ------------------------------------------------------------------------------------------
AT5G38540       ------------------------------------------------------------------------------------------
AT5G38550       ------------------------------------------------------------------------------------------
AT1G52120       ------------------------------------------------------------------------------------------
AT1G52130       ------------------------------------------------------------------------------------------
AT1G52110       ------------------------------------------------------------------------------------------
AT5G49850       ------------------------------------------------------------------------------------------
AT5G49870       ------------------------------------------------------------------------------------------
AT5G49860       ------------------------------------------------------------------------------------------
AT1G60130       ------------------------------------------------------------------------------------------
AT1G60095       ------------------------------------------------------------------------------------------
AT1G60110       ------------------------------------------------------------------------------------------
AT3G16460       ------------------------------------------------------------------------------------------
AT1G52030       ------------------------------------------------------------------------------------------
AT1G52040       ------------------------------------------------------------------------------------------
AT3G21380       ------------------------------------------------------------------------------------------
AT3G16430       ------------------------------------------------------------------------------------------
AT3G16420       ------------------------------------------------------------------------------------------
AT3G16440       ------------------------------------------------------------------------------------------
AT3G16450       ------------------------------------------------------------------------------------------
AT1G52100       ------------------------------------------------------------------------------------------
AT5G35950       ------------------------------------------------------------------------------------------
AT5G35940       ------------------------------------------------------------------------------------------
AT1G58160       ------------------------------------------------------------------------------------------
AT1G57570       ------------------------------------------------------------------------------------------
AT1G61230       ------------------------------------------------------------------------------------------
AT1G52050       ------------------------------------------------------------------------------------------
AT1G52060       ------------------------------------------------------------------------------------------
AT1G52070       ------------------------------------------------------------------------------------------
AT2G25980       ------------------------------------------------------------------------------------------
AT5G28520       ------------------------------------------------------------------------------------------
AT1G33790       ------------------------------------------------------------------------------------------
AT1G05760       ------------------------------------------------------------------------------------------
AT1G05770       ------------------------------------------------------------------------------------------
AT2G43730       ------------------------------------------------------------------------------------------
AT2G43740       ------------------------------------------------------------------------------------------
```

```
                640       650       660       670       680       690       700       710       720
            ....|....|....|....|....|....|....|....|....|....|....|....|....|....|....|....|....|....|
AT3G59590   ------------------------------------------------------------------------------------------
AT3G59610   ------------------------------------------------------------------------------------------
AT1G19715   ------------------------------------------------------------------------------------------
AT3G59620   ------------------------------------------------------------------------------------------
AT1G73040   ------------------------------------------------------------------------------------------
AT5G46000   ------------------------------------------------------------------------------------------
AT2G33070   ------------------------------------------------------------------------------------------
AT3G16390   ------------------------------------------------------------------------------------------
AT3G16410   ------------------------------------------------------------------------------------------
AT3G16400   ------------------------------------------------------------------------------------------
AT1G52000   TNSGNGGTNDGASGIGSNDGSTGTNPGAGGGTDSNIEGTENNVGGKETNPGASGIGNSDGSTGTSPEGTESNADGTKTNTGGKESNTGSE
AT2G39330   ------------------------------------------------------------------------------------------
AT2G39310   ------------------------------------------------------------------------------------------
AT3G16470   ------------------------------------------------------------------------------------------
AT5G38540   ------------------------------------------------------------------------------------------
AT5G38550   ------------------------------------------------------------------------------------------
AT1G52120   ------------------------------------------------------------------------------------------
AT1G52130   ------------------------------------------------------------------------------------------
AT1G52110   ------------------------------------------------------------------------------------------
AT5G49850   ------------------------------------------------------------------------------------------
AT5G49870   ------------------------------------------------------------------------------------------
AT5G49860   ------------------------------------------------------------------------------------------
AT1G60130   ------------------------------------------------------------------------------------------
AT1G60095   ------------------------------------------------------------------------------------------
AT1G60110   ------------------------------------------------------------------------------------------
AT3G16460   ------------------------------------------------------------------------------------------
AT1G52030   ---------------------------------------------------APA------------------------------------
AT1G52040   ------------------------------------------------------------------------------------------
AT3G21380   ------------------------------------------------------------------------------------------
AT3G16430   ------------------------------------------------------------------------------------------
AT3G16420   ------------------------------------------------------------------------------------------
AT3G16440   ------------------------------------------------------------------------------------------
AT3G16450   ------------------------------------------------------------------------------------------
AT1G52100   ------------------------------------------------------------------------------------------
AT5G35950   ------------------------------------------------------------------------------------------
AT5G35940   ------------------------------------------------------------------------------------------
AT1G58160   ------------------------------------------------------------------------------------------
AT1G57570   ------------------------------------------------------------------------------------------
AT1G61230   ------------------------------------------------------------------------------------------
AT1G52050   ------------------------------------------------------------------------------------------
AT1G52060   ------------------------------------------------------------------------------------------
AT1G52070   ------------------------------------------------------------------------------------------
AT2G25980   ------------------------------------------------------------------------------------------
AT5G28520   ------------------------------------------------------------------------------------------
AT1G33790   ------------------------------------------------------------------------------------------
AT1G05760   ------------------------------------------------------------------------------------------
AT1G05770   ------------------------------------------------------------------------------------------
AT2G43730   ------------------------------------------------------------------------------------------
AT2G43740   ------------------------------------------------------------------------------------------
```

```
              730       740       750       760       770       780       790       800       810
          ....|....|....|....|....|....|....|....|....|....|....|....|....|....|....|....|....|....|
AT3G59590 -----KP---------YEFAYD------------------WDF-----------MSSYSVSLNG--ALYWVAFHMASDDQFIQSFDFSTE
AT3G59610 ----------------TTMNASVTE--------LGSDA--WKSYEFAFHSWNLSMSPYSVSLNG--NLYWVAYNHESRDYFIQSFDFSTV
AT1G19715 -----TKHKLVTDTEKSQSKIEGGAKTYGPWGGTGGIM--FDD---------------GI-YTGIRQIN--LSRNVGIVSM--KVCY-DF
AT3G59620 ----------------SRDHADFVA--------HGGTE--WDD---------------GA-FVNVKKIK-IFHDWAGITLV--QFDYIDG
AT1G73040 ----------------VFVGPWGGN---------GGTT--WDD---------------GI-YDGVREIR-LVYD-HCIDSI--SVIY-DK
AT5G46000 -----EQ---------FRMPGTGGS---------NGVS--WDD---------------GA-YDRLSKLC-VGEDAHCVSSV--EFHY-VK
AT2G33070 -----SP---------ATPKLRGKW--------IKVEQ--KGE---------------GP---GPRCSHDIAQVGNKIFSF--GGELTPN
AT3G16390 -----LP---------STPKLLGNW--------IKVEQ--NGE---------------GP---GLRCSHGIAQVGNKIYSF--GGELIPN
AT3G16410 -----APLTNVTPLNAKKLEAKGGD---------TGDI--WDD---------------GV-YDNVRKVY-VGQAQYGIAFV--KFEY-VN
AT3G16400 -----LS---------STIKLLGKW--------IKVEQ--KGE---------------GP---GLRCSHGIAQVGNKIYSF--GGEFTPN
AT1G52000 SNTNSSP---------QKLEAQGGN---------GGNQ--WDD---------------GTDHDGVMKIH-VAVGGLGIEQI--RFDY-VK
AT2G39330 -----PPV--------YKLDAQGGT---------DGRV--WDD---------------GS-YDGVKSLR-IGQDNSRITYL--EFEY-EK
AT2G39310 -----TPFPL------YKLDAQGGT---------DGRV--WDD---------------GS-YDGIKTLR-IDQDNSRITYL--EVEY-EK
AT3G16470 -----TPLTPPVSGGLTKLEAQGGR---------GGDV--WDD---------------GGAYDNVKKVY-VGQGDSGVVYV--KFDY-EK
AT5G38540 -----TP---------NKLECQGDC---------RGMS--WDD---------------GCNYDGVRKVF-VDGIGNEIYTV--RFEY-DN
AT5G38550 -----TP---------NKLECQGDR---------KGLP--WDD---------------GCNYDGVKKVY--VDSISDIDSV--RFEY-DN
AT1G52120 -----TP---------TRMEAQGGN---------GGTK--WDD---------------GGDHDSVTKIQ-VRINKEGIQYI--KFNYVDK
AT1G52130 -----TP---------ARMEAKGGK---------GGNE--WDD---------------GGDYEAVTKIH-GRSDHKGIKDI--IFDYVDK
AT1G52110 -----TP---------TKLAWQGTA---------LGTL--WDH---------------GA-FQAVRKLS-VFEIGGYITCL--GITY-DN
AT5G49850 -----IP---------TKSECQGVT--------ERSTL--WDS---------------GA-FEGIRKVS-VTWRSYCIRCF--RINY-EN
AT5G49870 -----TP---------KKSECKGIT--------DGSNVWNWDD---------------GA-FEGIRKVS-VFFTALRIRCL--MINY-ED
AT5G49860 ----------------------------------------MDS---------------NV------------------------------
AT1G60130 -----TS---------PFIKLESRE--------STGDL--WDD---------------GT-FEGVRKVC-IHKQPVLYSVI--QFEY-VN
AT1G60095 -----SPLI-------SLEHTTGSD--------LVNHI--WDD---------------GS-FEGVRKVY-VRYDSLEICYV--EFDY-DN
AT1G60110 -----SPF--------TKLEV-GTT---------SADL--WDD---------------GT-FDGIRNVY-IHYDGDAVCCV--EVDY-DN
AT3G16460 -----TP---------LKLTAEGGE---------TGAV--WDD---------------GS-HDDVKKVY-VGQGQDGVAAV--KFEY-KN
AT1G52030 -----PP---------NKVEALGGN---------GGTI--FDD---------------GA-FDHVRKVY-IGQGDSGVAYV--KFEY-RK
AT1G52040 -----TP---------NKVEAQGGN---------GGET--FDD---------------GV-FDHVRKVY-VGQGESGVAYV--KFEY-EK
AT3G21380 -----SSSSSSSLSQANKVDAQGGK---------GGTS--WDD---------------GA-HDHVRRVY-IGQGDSGVTYV--KFEY-EK
AT3G16430 -----MA---------QKVEAKGGK---------GGNQ--WDD---------------GSDHDAVTKIQ-VAVGGMGIQYI--QFDY-VK
AT3G16420 -----MA---------QKVEAQGGK---------GANL--WDD---------------GSTHDAVTKIQ-LAAGIDGIQYV--QFDY-VK
AT3G16440 -----MA---------QKVEAQGGI---------GGDV--WDD---------------GA-HDGVRKVH-VGQGLDGVSFI--NVVY-EN
AT3G16450 -----MA---------QKVEAGGGA---------GGAS--WDD---------------GV-HDGVRKVH-VGQGQDGVSSI--NVVY-AK
AT1G52100 -----PS---------IQSAIEGAK--------TTGKG--FDD---------------GGDYDGIRKVY-VTTDGSAIRHV--RFDY-DK
AT5G35950 -----PA---------TKSAMDGGQ--------NTGRG--YDH---------------GGDYDGVRKVY-VTFDGTSIRNM--RVDY-DK
AT5G35940 -----LA---------TKSGMQGGQ--------NTGKS--YDY---------------GGDYDGIRKVY-VTYDGTSIRHM--RVDY-DK
AT1G58160 -----MA---------DKLEAKGGN---------GGKI--WDD---------------GV-HEG--------------------FDY-VK
AT1G57570 -----SP---------TKSECYGSS---------KGIY--WDD---------------GV-FDFIRTVY-VSSNVMNVRYI--KFHY-YN
AT1G61230 -----SV---------TKSVCHGSK---------IIES--WDD---------------GV-FDGIRKVY-VSYSINHVACI--TFEYISN
AT1G52050 -----AP---------TKSELQGGI--------TGGEY--WDD---------------GPNFDGVRKVY-VTFTETHIRSM--NIDY-DQ
AT1G52060 -----PP---------TKSELVGGW---------GGDY--WDD---------------GPNYDGVRKVY-VTYMNTCIRSI--NIDY-EK
AT1G52070 -----PP---------TKSQLVGGL--------TGGEP--WDD---------------GSNYDGVKKIS-VTYISTLIRSI--NVDY-EK
AT2G25980 -----PP---------IKQEYQGGT---------GGSP--WDH---------------GI-YTGIRKVY-VTFSPVSISHI--KVDY-DK
AT5G28520 -----GPIGLNPQVGHIKLAYQGGG---------GGIP--WDH---------------GPNHNGVKRVS-FIFDENEIRQW--RVDY-DD
AT1G33790 -----PP---------TKFDYQGGS---------GAQL--WDD---------------GTNYNGVRKIS-FALDANEIRQI--RIDY-DK
AT1G05760 -----------------KHDARSTT--------IVN----WDE---------------GSHDGFISQIF-LSHGVAGIMSI--QFQF-VM
AT1G05770 ----------------VGTDYSGKK---------TMVD--WDE---------------GSHNGIISQIF-LSHGPTGVFSI--QFQF-ML
AT2G43730 ----------------SKQYYDKS----------------WEE---------------KG-RNMISSIY-VAFNKDSIKCI--QFSYFHN
AT2G43740 ----------------QRAYLKNLE---------------WEE---------------KG-RNMISSIY-VAFDKDTINSI--QFSYCQN
```

```
                 820       830       840       850       860       870       880       890       900
             ....|....|....|....|....|....|....|....|....|....|....|....|....|....|....|....|....|....|
AT3G59590    KFEPYCLLPNKKCDPSNAR----------SLAV--FRG-DRFSY--LEQNYE-------TRNIEIWVTKK-EIKIENGKAVEWMNLMKVS
AT3G59610    SFKHYCILPTKNGHRQCDGRSLAIFR---EDRF-SFLEQEIYNTRNIEIWVTKETIKNGDGEAVEWVNLMSVLVPEWSSLSVNYYPPSYF
AT1G19715    RGQAVWG--SKHGGVGGFK-----HD---KIVF-DYPS-EVLTH--VTGTYG-----PLMYMGPNVIKSL-TFRTNRGKHGP-YG---EE
AT3G59620    TGAIVSG--LDHGTE---------------------PF---------------------------------QFDREHGRV--------PV
AT1G73040    NGKPAKS--EKHGGVGGNK-----TS---EIKL-QYPE-EYLTG--VSGYYC-----PMVNSGTPVIRSM-TFKSNKQVYGP-YG---VE
AT5G46000    GNDRI-T--HCHGKDSKEH------------------------------------------DKDGFISSL-TFKTSMNRSSEKFG---KP
AT2G33070    QPIDKHL--YVFDLETRTW----SIS---PATG-DVPNLSCLGV--RMVSIGSSLYVFGGRDASRKYNGFYSFDTTKNEWKLLTPVEQGP
AT3G16390    QPIDKHL--YVFDLETRTW----SIA---PATG-DVPHLSCLGV--RMVSVGSTLYTFGGRDFSRQYNGFYSFDTTTNEWKLLTPVEEGP
AT3G16410    GSQVVVG--DEHGKKTELG-----VE---EFEI-D-AD-DYIVY--VEGYRE-----KVNGMTSEMITFL-SFKTYKGKTSQPIE---QR
AT3G16400    QPIDKHL--YVFDLETRTW----SIS---PATG-DVPHLSCLGV--RMVSVGSTLYVFGGRDASRQYNGFYSFDTTTNEWKLLTPVEEGP
AT1G52000    NGQLKEG--PFHGVKGRGG-----TS---TIEI-SHPD-EYLVS--VEGLYD----------SSNIIQGI-QFQSNK-HTSQYFGYEYYG
AT2G39330    GGKLET---CRHGVKQERS---------LKFEL-N-PD-EYIKS--VEATYD-----KPDIFRNVVITSL-TFETSKGRTS-FSG---YK
AT2G39310    DGEAKT---CNHGGKGDTP------S---EFVL-GYPD-EYIKS--VEATYQ-----KPNIFSNTAITSL-KFLTSKGRTS-FFG---YN
AT3G16470    DGKIVS---LEHGKQTLLG-----TE---EFEI-D-PE-DYITY--VKVYYE-----KLFGSPIEIVTAL-IFKTFKGKTSQPFG---LT
AT5G38540    GGKVEKT--PYRRDVKNEK----------EFVL-DYPN-EFITS--VEGTLA-----APKSVNITWITSL-TFKTSKKRSSPTFG---SA
AT5G38550    GGKVEKT--PYRRDVTNEK----------EFVL-DYPN-EFITS--VEGTLA-----TPTNFDITWILSL-TFKTSKGRTSPTFG--SSS
AT1G52120    DGDPEKE--QLHGSETGRG---YTLE---PFEI-NHSDKEYLLS--IDGCYD---------EDSGVIQSL-QLKTNI-KTSEVMG--DDE
AT1G52130    DGHPKS---ETHGPTSGQG---YVLE---PFEI-NHLDKEYLMS--IDGYYD---------DASGVIQAL-QFKTNM-KTSELMGYYDDD
AT1G52110    DGKVEK---RDHGMQDGYP---GEEV---EFVV-DYPN-EFITS--VVGTMS-----------TDRVASL-TFKTSKGRTSQRFGDRTAN
AT5G49850    DGKVVK---RAHGMNDDSR----ITD---EFVV-DYPY-EVITS--IVGTMN-----------DSYVTSF-VFKTSKGRTSRTFGERTSD
AT5G49870    AGKVVK---RSHGLNNNDH----QEE---KFVV-DYPN-EFITS--VVGTMS-----------SSSVMPL-IFKTSKGRTSKQFGDYSFD
AT5G49860    ------------------------------------------------------------------ITSL-TFKTSKGRTSPKFG-YGTS
AT1G60130    RGEVEN---RDLGLRVFIA----EEG---EFVV-NYPY-EFIIS--VEGTFT--------NEKDPHVASL-TFKTSKGRTSSTFG---TP
AT1G60095    KGKVEK---REHGMFYSWV----QQG---EFVV-DYPN-EFITS--VEGTMR--------TESFMQVASL-TFKTSKGRTSSTFG---SP
AT1G60110    KGKVEK---REHGIMIAPFIERG------EFVV-DYPN-EFITS--VEVTISKQNDSPVPSLTSETVASL-TFKTSKGRTSSTFG---SP
AT3G16460    GSQVVFG--DERGTRTLLG-----FE---EFEL-E-SD-EYITS--VEGYYE-----KNFGVDT-VVTTL-IFKTSKNKTAGPFG---IV
AT1G52030    DGKRET---REHGKMTVLG-----TE---EFEV-E-SD-DYITS--IEVSVD-----NVFGFKSEIVTSL-VFKTFKGITSQPFG---ME
AT1G52040    DGKRET---RDHGKMTLLG-----TE---EFEV-D-SD-DYITS--IEVSVD-----KVFGYNSEIVTAL-VFKTSKGTTSPPFG---MV
AT3G21380    DDKKES---REHGKKTLLG-----AE---VFEV-D-PD-DYITS--VEVQSD-----RIFGQDTEVITSL-IFKTSKGKFSPPFG---LE
AT3G16430    NGQTEQT--PLRGIKGSTI----PTD---PFVI-NHPE-EHLVS--IEIWYK----------PDGLIQGL-RFISNK-KTSRFIG---YD
AT3G16420    NGQPEQA--PLRGTKGRVL----PAD---PFVI-NHPD-EHLVS--VEGWYS----------PEGIIQGI-KFISNK-KTSDVIG---SD
AT3G16440    GSQEVVG--GEHGKKSLIG-----IE---TFEV-DADD--YIVA--VQVTYD-----KIFGYDSDIITSI-TFSTFKGKTSPPYG---LD
AT3G16450    DSQDVEG--GEHGKKTLLG-----FE---TFEV-D-AD-DYIVA--VQVTYD-----NVFGQDSDIITSI-TFNTFKGKTSPPYG---LE
AT1G52100    AGQVET---RERGAKTGTQ------H---EFTV-NHPY-EYITS--VEGTYA-----HTQPYNCVVLTSL-TFKTSKGRASPAIG---KV
AT5G35950    VGQVEC---YEYGVKIGTQ------N---QFTI-NYPY-ECITS--VGGSYA-----DTQPYRCIVLRSL-TFKTSNGRTS-VFG---KE
AT5G35940    AGQVEC---YEYGDKTGTQ---------YKITV-NYPY-ECITS--VEGSYA-----NTQPYGCIVLRSL-TFKTSNGRT-LVIG---TV
AT1G58160    NGQPKAG--STHGVSYHNF-----TE---WFDL-NHTCDEHILS--VKCYYD-----------DGEIQGL-VIKTNI-RTSAYMG---YN
AT1G57570    RAVVV----RQHGWNSIVE--EDGEK---EFEL-DYPN-ELITS--VEGTMK------SFSRSEIRISSL-TFKTSKGRTSPTIG---IA
AT1G61230    HSVVK----RQHGNNTSLV------E---EFEL-NYPN-EFITS--VDGTFK-----NSGMRKVMCVTSL-VFKTSKGRISPTYG---SV
AT1G52050    DGQVVT---RYHGMKNGET------Q---EFAV-DFPN-EYMTS--VEGTYD-----HISEGNYLVLTSL-TFKTSKGRISQTFG---LV
AT1G52060    DGQVVT---SSHGNKEGET------E---EFAI-DYPN-EFLIS--VEGTYD---SILFPDHYVLVITSL-SFKTSKGRISPTYG---VV
AT1G52070    DGQVVT---RYHGMKNGDT------E---EFVI-DYPN-EYLIS--VEGTYN-----ILPDDNVLVIRSL-IFKTSKGRISPTYG---FV
AT2G25980    DGKVET---RQDGDMLGENRVQGQPN---EFVV-DYPY-EYITS--IEVTCD------KVSGNTNRVRSL-SFKTSKDRTSPTYG---RK
AT5G28520    GGVI-----RQYEPINGYD----------MFEVKEYPT-EYIIS--VECTYD----DVIPRSGRRMIRSI-MFKTSKGRVSPIFG---YP
AT1G33790    GGLIER---REYGGNVGRQ------E---EFVV-DYPS-EYIIY--MEGTCD-----IVSDASKNRVRSL-MFKTSKGRTSPIFG---KV
AT1G05760    DGKLVLS--DRHGPFSGNM-----FD---VIEL-NYPH-EYITG--ISGEYY------KYEANNPHMRSL-KFNTNTSEYGP-FGTSGSS
AT1G05770    DDTFFLS--SCHGQNTGSM-----FD---VILL-NCPH-EYITG--ISGEYL-----KSDGASGPQIRSL-AFATNLNQYGPFGG--SSS
AT2G43730    GVHVVS---EKHGSSKGQS----------YEIV-RLNDDEYVTG--LSGIFW-----------ERKVTSL-TFHTNQGKHGPFCNGTGYS
AT2G43740    GGHVVS---KKYGMSDAQKQKYGSYNGRFHVMV-RLNDDEFVTG--LSAIYL-----------CKGITNL-NIHTNQGKHGPICDRYSSS
```

```
                910       920       930       940       950       960       970       980       990
          ....|....|....|....|....|....|....|....|....|....|....|....|....|....|....|....|....|....|
AT3G59590 VPKWSSLREKACIYIAKGDK---FHEIIINDLVEYPTRHHRT--YVPS------------------------------------------
AT3G59610 VDEDKVGLTLVICCYNKEGKAYIYIAKGDKFHEIEIKDLVEY---NPR------------------------------------------
AT1G19715 QG------PSFTHQ-MDEGKVVGFLGREGL----FLDSIGVHVMECKI------------------------------------------
AT3G59620 EG------ARAEFWLRDDEYI------------TIVTAFGGY------------------------------------------------
AT1G73040 QG------TPFTFS-VNGGRIVGMNGRSGW----YLDSIGFH------------------------------------------------
AT5G46000 VG------TKFQLEAKGFDKIVGFRGRSSV---NRINALGAN--FAVV------------------------------------------
AT2G33070 TP------RSFHSMTADENNVYVFGGVSAT---VRLKTLDAY--NIVDH-----------------------------------------
AT3G16390 TP------RSFHSMAADEENVYVFGGVGAM---DRIKTLDSY--NIVDK-----------------------------------------
AT3G16410 PG------IKFVLQ---GGKIVGFHGR-ST---DVLDSLGAYISLSPTPNLHGKWTKVDENGDGPGLRCSHDIAQVGNKIYSFGGEFTPN
AT3G16400 TP------RSFHSMAADEENVYVFGGVSAT---ARLNTLDSY--NIVDK-----------------------------------------
AT1G52000 DG------TQFSLQ-VNEKKIIGFHGFADS----HLNSLGAY--FVPI-S----------------------------------------
AT2G39330 GG------KKFKLE-QKGRRLVGFHGKEGS----AIDALGAY--FAPI------------------------------------------
AT2G39310 VG------KKFVLE-QKGHRLVGFHGKEDA----AIDALGAY--FGPV------------------------------------------
AT3G16470 SG------EEAELG---GGKIVGFHGTSSD----LIHSLGAY--IIPS------------------------------------------
AT5G38540 SS------RKFVLE-KNGSPLVGFHGYNSV--GNTLNSLGAY--YRPI------------------------------------------
AT5G38550 PG------RKFVLE-KNGSALVGFHGYIGP--GYNIKALGAY--YRPI------------------------------------------
AT1G52120 KG------TKFTLG-CNGHEIIGFHGSAQD----NLNALGAY--ITTLT-----------------------------------------
AT1G52130 A-------VKFTIG-CTVNKIIGFHGHAGK----NLYSLGAY--FTTL------------------------------------------
AT1G52110 L-------VEFVLE-NKDCAIVGFHGWWTP---SYLTALGAY--SFPM------------------------------------------
AT5G49850 S-------VEFVIE-SKGCAVVGFHGWYAPLGAGYITALGAH--FYPM------------------------------------------
AT5G49870 DS------VEFVLE-SKGCAIVGFHGWHNP-VSGYMTALGAY--YYPM------------------------------------------
AT5G49860 DS------VEFVLE-SKGCAIVGFYGWYKT-GSGYTTALGAY--YYPM------------------------------------------
AT1G60130 GT------KKFVLQ-SKGCGVVGFHGVLSN---DYISGLGAY--FRLL------------------------------------------
AT1G60095 SD------SKFLLE-SKGCGVVGFYGRCFS----SIFDLGAY--FRPL------------------------------------------
AT1G60110 AT------KKFVLQ-SKGCGVVGFLGRSSY----YTYALGAH--FCPL------------------------------------------
AT3G16460 SG------TKFEFK-KEGYKITGFHGR-AG---EYVNAIGAY--LAPS------------------------------------------
AT1G52030 TE------KKLELKDGKGGKLVGFHGK-AS---DVLYALGAY--FAPTT-----------------------------------------
AT1G52040 TE------KKFELKDGNGGKLAGFHGK-AS---DVLYALGAY--FAPS------------------------------------------
AT3G21380 GS------QKYELKDKNGGKLVGFHGRVGG---ELLNALGAY--FAPS------------------------------------------
AT3G16430 RG------TRSFLQ-VQDKKIIGFHGSAGD----NLNSLGAY--FAPL------------------------------------------
AT3G16420 EG------THFTLQ-VKDKKIIGFHGSAGG----NLNSLGAY--FAPL------------------------------------------
AT3G16440 TE------NKFVLKEKNGGKLVGFHGRAGE----ILYALGAY--FTTT------------------------------------------
AT3G16450 TQ------KKFVLKDKNGGKLVGFHGRAGE----ALYALGAY--FATT------------------------------------------
AT1G52100 TG------SKFKLE-RQGDAIVGFHGR-VG---SCIDGIGVY--YAPL------------------------------------------
AT5G35950 TG------TTFLLE-SQGNAIVGFHGRVGS----CVDSIGEY--YAPF------------------------------------------
AT5G35940 TG------TKFLLE-SKGNAIVGFHGRVGS----CVDSIGAY--YAPF------------------------------------------
AT1G58160 IG------TTFTLE-VKGKKIVGFHGSFDK----NLTSLGAY--FAPL------------------------------------------
AT1G57570 SG------TKFLLA-SKGCAVVGFYGRHDD---RDLVAIGAY--FSPL------------------------------------------
AT1G61230 TG------TKFVLE-TKDCALAGFHGWTFL---GFLTAIGAY--FSLL------------------------------------------
AT1G52050 IG------TKFVLE-TKGNVISGFHGRDGG----SFDAIGVY--FSPM------------------------------------------
AT1G52060 SG------TKFVLE-SQGNAIVGFYGRNGG----AFDAIGVY--FSPI------------------------------------------
AT1G52070 SG------TKFVLE-SQGNAIVGFYGRDGG----AFDAIGVY--FSPI------------------------------------------
AT2G25980 SE------RTFVFE-SKGRALVGLHGRCC----WAIDALGAH--FGAP------------------------------------------
AT5G28520 AA------RKFVLE-NNGGALIGFHGRVGA----GIDALGAY--FSSF------------------------------------------
AT1G33790 AA------RKFVFE-SNGSALIGFHGRAAA----AVDAIGAY--FSRF------------------------------------------
AT1G05760 ND-------KFAFKLGKSPQFGGFHGTYDA---SGLQYIGVY--LRPK------------------------------------------
AT1G05770 QSSIWNHEQQFRFKLGKFRQFSGFYGTYNA---SGLQNIGVY--LQPT------------------------------------------
AT2G43730 SSYMADYKKEIDVKIRDRREFGGFFGSFDD-EFDGLTSIGIY--VYPT------------------------------------------
AT2G43740 KNIMDNYKVEIDVKIRDRREFGGFFGSFDN--YGTLTSIGIY--VCPI------------------------------------------
```

```
                1000      1010      1020      1030      1040      1050      1060      1070      1080
            ....|....|....|....|....|....|....|....|....|....|....|....|....|....|....|....|....|....|
AT3G59590   ------------------------------------------------------------------------------------------
AT3G59610   ------------------------------------------------------------------------------------------
AT1G19715   ------------------------------------------------------------------------------------------
AT3G59620   ------------------------------------------------------------------------------------------
AT1G73040   ------------------------------------------------------------------------------------------
AT5G46000   ------------------------------------------------------------------------------------------
AT2G33070   ------------------------------------------------------------------------------------------
AT3G16390   ------------------------------------------------------------------------------------------
AT3G16410   QPIDKHLYVFDIESRTWSISPATGDIPTLSCLGVCMVSIGSTLYVFGGRDASRQYNGFYSFDTTTNEWKLLTPVEEGPTPRSFHSMAADE
AT3G16400   ------------------------------------------------------------------------------------------
AT1G52000   ------------------------------------------------------------------------------------------
AT2G39330   ------------------------------------------------------------------------------------------
AT2G39310   ------------------------------------------------------------------------------------------
AT3G16470   ------------------------------------------------------------------------------------------
AT5G38540   ------------------------------------------------------------------------------------------
AT5G38550   ------------------------------------------------------------------------------------------
AT1G52120   ------------------------------------------------------------------------------------------
AT1G52130   ------------------------------------------------------------------------------------------
AT1G52110   ------------------------------------------------------------------------------------------
AT5G49850   ------------------------------------------------------------------------------------------
AT5G49870   ------------------------------------------------------------------------------------------
AT5G49860   ------------------------------------------------------------------------------------------
AT1G60130   ------------------------------------------------------------------------------------------
AT1G60095   ------------------------------------------------------------------------------------------
AT1G60110   ------------------------------------------------------------------------------------------
AT3G16460   ------------------------------------------------------------------------------------------
AT1G52030   ------------------------------------------------------------------------------------------
AT1G52040   ------------------------------------------------------------------------------------------
AT3G21380   ------------------------------------------------------------------------------------------
AT3G16430   ------------------------------------------------------------------------------------------
AT3G16420   ------------------------------------------------------------------------------------------
AT3G16440   ------------------------------------------------------------------------------------------
AT3G16450   ------------------------------------------------------------------------------------------
AT1G52100   ------------------------------------------------------------------------------------------
AT5G35950   ------------------------------------------------------------------------------------------
AT5G35940   ------------------------------------------------------------------------------------------
AT1G58160   ------------------------------------------------------------------------------------------
AT1G57570   ------------------------------------------------------------------------------------------
AT1G61230   ------------------------------------------------------------------------------------------
AT1G52050   ------------------------------------------------------------------------------------------
AT1G52060   ------------------------------------------------------------------------------------------
AT1G52070   ------------------------------------------------------------------------------------------
AT2G25980   ------------------------------------------------------------------------------------------
AT5G28520   ------------------------------------------------------------------------------------------
AT1G33790   ------------------------------------------------------------------------------------------
AT1G05760   ------------------------------------------------------------------------------------------
AT1G05770   ------------------------------------------------------------------------------------------
AT2G43730   ------------------------------------------------------------------------------------------
AT2G43740   ------------------------------------------------------------------------------------------
```

```
                1090      1100      1110      1120      1130      1140      1150      1160      1170
          ....|....|....|....|....|....|....|....|....|....|....|....|....|....|....|....|....|....|
AT3G59590 ----------------------------LVPVPT------------------FTMSNRSKTQQVESRFTPPRGIQ----------ASDG-
AT3G59610 ----------------------------HRTYFP------------------NLIQVPTFTMSGRSITQHQVESRFAPLRGIQVSVGVG-
AT1G19715 --------------------------SSLKPSSPHNAIVPHNNSGTAQIENSPWANKLVLAANGHGEEVDRGVVKEPTPSGPGPWGGDG-
AT3G59620 ------------------------------------------------------------------------------------------
AT1G73040 --------------------------------------------------------------------LSRPKST---------------
AT5G46000 ------------------------------VVPP----------------------------------VKKLNAK----------GGVL-
AT2G33070 --------------------------KWVQCSTP----------------------------------GGSCSVR----------GGAGL
AT3G16390 --------------------------TWFHCSNP----------------------------------GDSFSIR----------GGAGL
AT3G16410 ENVYVFGGVSATARLNTLDSYNIVDKKWFHCSTP----------------------------------GDSLTAR----------GGAGL
AT3G16400 --------------------------KWFHCSTP----------------------------------GDSLTAR----------GGAGL
AT1G52000 --------------------------SSSSSLTP------------------P---------------PNKVKAQ----------GGSY-
AT2G39330 --------------------------PTPTPIIP----------------------------------AKKLPAI----------GGSG-
AT2G39310 --------------------------PTPTPLIP----------------------------------SKKLPAI----------GGNE-
AT3G16470 ----------------------------STPLTP------------------S---------------SNTIPAQ----------GGDG-
AT5G38540 ------------------------------PPTP------------------D---------------VEKLKAQ----------GGVE-
AT5G38550 ------------------------------PPTP------------------D---------------VKRLEAQ----------GGDG-
AT1G52120 --------------------------------------------------------------------LTKLEYI-----------GEG-
AT1G52130 ------------------------------PLTK------------------LEYEDSF---------REKLPKN----------GAS--
AT1G52110 ------------------------------PLSP------------------A---------------SEKLEAQ----------GGDG-
AT5G49850 ------------------------------PLPP------------------A---------------AEKLEAQ----------GGAG-
AT5G49870 ------------------------------PLPP------------------A---------------AEKLEAQ----------GGAG-
AT5G49860 ------------------------------PLPP------------------S---------------SEKLEAQ----------GGAG-
AT1G60130 ------------------------------PPLP------------------D---------------GEKVEAK----------GGDG-
AT1G60095 ------------------------------PPPS------------------N---------------TEKVEAK----------GGDG-
AT1G60110 -----------------------------PPLPD----------------------------------GEKVEAK----------GGDG-
AT3G16460 ---------------------------GTTPLTP------------------ATQ-------------SQKLEGA----------GSEA-
AT1G52030 --------------------------NSTTPSTP------------------ST--------------SKKLQAR----------GGNG-
AT1G52040 ----------------------------TTSTTP------------------ST--------------TKKLQAR----------GGNG-
AT3G21380 ----------------------------SGRGTP------------------SATQPPGSAQPTGSAGAKKLEAK----------GGNV-
AT3G16430 ----------------------------TIPLTP----------------------------------AKPLPAL----------GSDD-
AT3G16420 --------------------------TTTTPLTP----------------------------------AKQLTAF----------GSDD-
AT3G16440 ----------------------------TTPLTP----------------------------------AKKLPAV----------GGDE-
AT3G16450 ----------------------------TTPVTP----------------------------------AKKLSAI----------GGDE-
AT1G52100 -----------------------------PPSPP------------------P---------------PEKLQGQ----------GGDG-
AT5G35950 -----------------------------SPYPP------------------P---------------TEKLEGQ----------GGDG-
AT5G35940 -----------------------------SPSPP------------------P---------------TQKLEGQ----------GGDG-
AT1G58160 ------------------------------------------------------------------------------------------
AT1G57570 -------------------------------PPP------------------T---------------AEKLQAQ----------GGNQ-
AT1G61230 ------------------------------PCPP------------------N---------------AEKLEAR----------GYDR-
AT1G52050 ------------------------------------------------------------------------------------------
AT1G52060 ------------------------------------------------------------------------------------------
AT1G52070 ------------------------------------------------------------------------------------------
AT2G25980 ----------------------------PIPPPP------------------P---------------TEKLQGS----------GGDG-
AT5G28520 ---------------------------IPSPPPP------------------S---------------PEKLQPE----------GGEAV
AT1G33790 ------------------------------ILPP------------------S---------------AETLQAK----------GGEG-
AT1G05760 ------------------------------------------------------------------------------------------
AT1G05770 ------------------------------------------------------------------------------------------
AT2G43730 --------------------------------------------------------------------YDNKPTL---------------
AT2G43740 ------------------------------------------------------------------------------------------
```

```
                  1180      1190      1200      1210      1220      1230      1240      1250      1260
            ....|....|....|....|....|....|....|....|....|....|....|....|....|....|....|....|....|....|
AT3G59590   ----GNEWDDGI-FHNVKKINVGVNDFDTVFVKFHYSKY-NRIEAGAGHGNATTHNPDDE---IMIAGGDYIEA----------VEGTYT
AT3G59610   ----GDEWDDGF-FDNVKEIIIHTNSLGIIFVKFYYRNG-NVRVAGAAHGDSTETR------GLMVPDDDYIEA----------VQGTYT
AT1G19715   ----GQAWDDGV-FSGIKQIFVTRGNDAITSIQIEYDRNGQSVWSIKHGGDSNGVATHR---IKFEYPDESITC----------ISGYYG
AT3G59620   ------------------------------------------------------------------------------------------
AT1G73040   ------------------------------------------------------------------------------------------
AT5G46000   ----GNEWDDGI-HDDARMIT-----------------------------------------FKLYFNKEYITS----------VEGHYG
AT2G33070   EVVQGKVWVVYG-FNGCE-------VDDVHCYDPAQDKWTQVETFGEKPCARSVFASAVVGKHILVFGGEIAMDPKAHEGPGQLSGGTFA
AT3G16390   EVVQGKVWIVYG-FNGCE-------VDDVHFYDPAEDKWTQVETFGVKPNERSVFASAAIGKHIVIFGGEIAMDPRAHVGPGQLIDGTFA
AT3G16410   EVVQGKVWVVYG-FNGCE-------VDDVHYYDPVQDKWTQVETFGVRPSERSVFASAALGKHIVIFGGEIAMDPLAHVGPGQLTDGTFA
AT3G16400   EVVQGKVWVVYG-FNGCE-------VDDVHYYDPVQDKWTQVETFGVRPSERSVFASAAIGKHIVIFGGEIAMDPLAHVGPGQLTDGTFA
AT1G52000   ----GETFDDGA-FDHVRKVYVGQGDSGVAYVKFDYEKDGKKET--QEHGKMTLSG-TEE--FEVD-SDDYITS----------MEVYVD
AT2G39330   ----GVAWDDGV-YDGVRKILVGQGNDGVAFVKFEYNKG-KDLVSGDDHGKMTLLG-TEE--FVLE-DGEYLTA----------IDGYYD
AT2G39310   ----GVTWDDGV-YDGVRKILVGQGNDGVSFVKFEYSKG-KDLVPGDDHGKKTLLG-AEE--FVLE-DGEYLMN----------IDGYYD
AT3G16470   ----GVAWDDGV-HDSVKKIYVGQGDSCVTYFKADYEKA-SKPVLGSDHGKKTLLG-AEE--FVLG-PDEYVTA----------VSGYYD
AT5G38540   ----------------------------------------------------ELLG-MMV--FELD-PLERITS----------VEGTYD
AT5G38550   ----GASWDDGGTFNSVRKIYIGLGKNVVGFVKFLYYKN-ARVVIGDDHGNKTLSSDLLE--FLLD-PFEHIIS----------VEGTYD
AT1G52120   ----SDIWDDGT-FEGVKKVSFYHNDGIVRCIEFDYVKD--GKIETRVQGGKRGTGDFTKEEFTVDYPNEFLTS----------VEGTYR
AT1G52130   ----GNLWDDGS-FQGVKKVHIYYDGYSVRCVRFDYDDD--GKVESREHGPKIVAA-VQEGGFVLDYPNEVITS----------VEGIAT
AT1G52110   ----GASWDDGGNFEGVRKICIGTGEIGIVSVKFLYENDTHEIVVGDHHGNKNLIK-HEE--FELDYPGEYLTL----------VEGSYD
AT5G49850   ----GVPWDDGSNFERVRKIYIGTCEVGIVSVRFLYENDIEEIVVGDHHGNKNLLR-HEE--FDLDNACEYLTS----------VEGSYD
AT5G49870   ----GAPWDDGSNFQGVRKIYIGTGEVGIVSIKFLYENDVHEIVVGDHHGNKNLLR-HEE--FDLDYPSEYLTS----------VEGSYD
AT5G49860   ----GAPWDDGSNFEGVRKIYIGTGEIGIVSIKFLYENDIHEIIVGDHHGNKNLLR-HEE--FDLDYPSEYLTS----------VEGSYD
AT1G60130   ----GASWDDGG-FDCIRKIYIGHGEMGIAFVKFLYDKD-NKFVVGDDHGSKTLLG-VDE--FELEHPDEYLIS----------VEGSYD
AT1G60095   ----GASWDDGG-FDGIRNIYIGHNKMGIAFVKFLYDKD-SQIVVGDDHGSNTLLR-VDE--FELEHPGEYLIS----------VEGSYD
AT1G60110   ----GASWDDGR-FDCIRKIYIGHSEMGIAFVKFLYDKD-NKVVVGDDHGSKTLLG-VDE--FELEHPDEYLIS----------VEGSYD
AT3G16460   ----GTLWDDGA-FDGVRKVSVGQAQDGIGAVSFVYDKA-GQVVEGKEHGKPTLLG-FEE--FELDYPSEYITA----------VDGTYD
AT1G52030   ----GASWDDGV-FDGVRKILVGQGNDGVAFVTFEYNKG-SQAILGDRHGKQTLLG-TET--FELDYPSEYITS----------VEGYYD
AT1G52040   ----GASWDDGV-FDGVRKILVGQGNDGVAFVTFEYNKG-SQAILGDGHGKKTLLG-TET--FELDYPSEYITS----------VEGYYD
AT3G21380   ----GNPWDDGP-HEGVRKVYIGQGDSGVSYVKFVYDKD-SKEVPGNDHGKRTLLA-PEE--FLLEYPNEYITS----------VELNYD
AT3G16430   ----GTAWDDGA-YVGVKKVYVGQAQDGISAVKFVYDKS-PEEVTGEEHGKSTLLG-FEE--FVLDYPSEYIIA----------VEGTYD
AT3G16420   ----GTVWDDGA-YVGVKKVYVGQAQDGISAVKFVYDKS-PEEVTGEEHGKSTLLG-FEE--FVLDYPSEYITA----------VDGTYD
AT3G16440   ----GTAWDDGA-FDGVKKVYIGQAQDGISAVKFVYDKG-AEDIVGDEHGNDTLLG-FEE--FQLDYPSEYITA----------VEGTYD
AT3G16450   ----GTAWDDGA-YDGVKKVYVGQGQDGISAVKFEYNKG-AENIVGGEHGKPTLLG-FEE--FEIDYPSEYITA----------VEGTYD
AT1G52100   ----GDSWDDGA-FKNVKKIYVGQGDVGIAAVKFEYETYTSEVILAERHGKETLLG-YED------------------------------
AT5G35950   ----GDSWDDGA-FLNVKKVCIGQGQFGIVSVKFEYENDASEVVVGDEHGKATLLG-YEE--FELDYPSEYITS----------VEACQD
AT5G35940   ----GDSWDDGA-FLNVKKVYIGQGSNGIVAVKFEYENDASEVVVGDEHGKTTLLG-YEV--FELDYPNEYITS----------LEGCHD
AT1G58160   ------------------------------------------------------------------------------------------
AT1G57570   ----GDSWDDGV-FEGVRKLYVGQGKNCVAFLKVVYDSN-TQVVIGEDHGNKTLFE-VKE--YELEYPSEYITA----------VDGCYN
AT1G61230   ----GAFWDDGV-YDGVRKIYVGQCENGIAFLKFVYDKD-TRMVIGDDHGNKTPLE-VKE--LDLEYPGEYVTA----------VEGCYN
AT1G52050   -----------------------------------------------------------------------ISS----------------
AT1G52060   ------------------------------------------------------------------------------------------
AT1G52070   ------------------------------------------------------------------------------------------
AT2G25980   ----GESWDDGA-FDGVRKIYVGQGENGIASVKFVYDKN-NQLVLGEEHGKHTLLG-YEE--FELDYPSEYITA----------VEGYYD
AT5G28520   ----GDPWDDGI-FNGVREIHLEDGEG--IALKFVYDKD-VQVTELKVHGEPSGIG-FNE--FKLDYPSEYITT----------VEGFWD
AT1G33790   ----GDPWSDGV-FNGVRNIYVGQGENGVSAVKFVYDKD-SQVAEGNDHGKPTLLG-YEE--FKLEYPSEYITT----------VEGCFD
AT1G05760   ------------------------------------------------------------------------------------------
AT1G05770   ----------------------------------------IVKPTGTRNAEET-------------------------------------
AT2G43730   ----NQAWDP---LYAFAHNQIPTIADGIPVMHIRYKPKLKDRIF-----------------SKLDFKRAMLCL----------IKVFLD
AT2G43740   ---------------------TRINDVALRTNYKVTDDYDDQSTFYQSSGPLTTINHNRK--LEYQMPHEVSDV----------KPIVHN
```

```
                 1270      1280      1290      1300      1310      1320      1330      1340      1350
           ....|....|....|....|....|....|....|....|....|....|....|....|....|....|....|....|....|....|
AT3G59590  E-----------------SHITSITFRMRKGDM------MPQYGRLNG---TPFSLRGERGSKAIGFYGRSSGVHLTALGVHFSPPPLYY
AT3G59610  E-----------------SHITSMAFRLHKGNR------SLRFGFFEG---MSFVLGGARGSKIIGFYGRSSDLYLTAFGVHFSPLP---
AT1G19715  PLNN----SDR------YNVVKSLSFYTSRGR-------YGPYGEETG---TFFTSTT-TQGKVLGFHGRSSFH-LDAIGVHMQHWLGNN
AT3G59620  -------------------CVRKIEFITNK-RT-----------FDFG-----------------GFN----------------------
AT1G73040  ------------------KMINKLRKKIHW---------------------------------------------LTRIVA---------
AT5G46000  KRLAAPNASASAMSSFFTGYMTMLKFNTNR-TTYQVLSHSPEYTYEG----TSFKLEE-KDHKIVGFYGKTEVS-LNQIGVYVKPIANA-
AT2G33070  LD------TET------LKWEKLDKLGEEE-ET------PSIRGWSAS---TTGTIDGKKGLVMFGGKAQTNDR-FGDLFFYGVDSA---
AT3G16390  LDTE------------TLQWERLDKFEG----T------PSSRGWTAS---TTGTIDGKKGLVMHGGKAPTNDR-FDDLFFYGIDSV---
AT3G16410  LDTE------------TLQWERLDKFGGEE-ET------PSSRGWTAS---TTATIGGKKGLVMHGGKAPTNDR-FDDLFFYGIDSA---
AT3G16400  LDTE------------TLQWERLDKFGGEE-ET------PSSRGWTAS---TTATIDGKKGLVMHGGKAPTNDR-FDDLFFYGIDSA---
AT1G52000  KVYG----YKS-------EIVIALTFKTFKGET------SPRFGIETE---NKYEVKDGKGGKLAGFHGKASDV-LYAIGAYFIPAAN--
AT2G39330  KIFG----VET-------PMIICLQFKTNK-RE------STPFGMDSG---EKFSLGE-KGHKIVGFHGQASDV-VHSIGVTVVPITNTK
AT2G39310  KIFG----VEE-------PIIVCLQFKTNK-RE------SMPFGMDSG---KKFSLGE-EGHKIVGFHGQASDV-VHSIGVTIVPITTTE
AT3G16470  KIFS----VDA-------PAIVSLKFKTNK-RT------SIPYGLEGG---TEFVLEK-KDHKIVGFYGQAGEY-LYKLGVNVAPIAK--
AT5G38540  DKIG---------------GITMLRFKTNK-KD------SPYFGFGTL---PSFVLHK-DNHQIVGFHGKSSNM-LHQLGVHVLPNGFKF
AT5G38550  DTSG---------------GITMLRFETNL-QK------SPYFGFGTT---SNFLLHK-DNHQIVGFHGKSSNM-LHQLGVHVIPNGFKF
AT1G52120  DNPG-------------GTLITSLTFKTSNNRT------SPILGKASN---KTFLLES-KGCALVGFHGASSDFFLYALGAYSFPMTSLA
AT1G52130  VVNTGLSFSTG------NVMIKSLTFKTSKGRT------SPTFGNVFGNYLSEFKLES-QGCAIVGFHGRSSYNSIHGLGAYFFPMPPSH
AT1G52110  IVPG----SEE------TEVIIMLMFTTNM-RA------SPCYGLDDN---PSFVLQK-RGHKIVGFHGKSSKM-LHQLGIHVLPITHP-
AT5G49850  VIPG----SED------VEVILMLKFTTNK-RT------SPCYGLDDD---PTFVLHK-AGHRIIGFHGKSSNM-LHKLGIHVLPITDP-
AT5G49870  VVPG----SEE------YEVIIMLKFTTNK-RT------SPCYGLDDD---PIFVLHK-EGHKIVGFHGKSSNM-LHKLGIHVLPISNS-
AT5G49860  VVPG----SEE------DEVMIMLKFTTNM-RT------SPCYGLDDD---PSFVLHK-EGHKIVGFHGKSSTM-LHKLGIHVLPITHS-
AT1G60130  VVDG----SES-------EVILMLRFKTNM-RT------SQVFGLDTT---SSFILEK-ECHKIVGFHGKIGKM-LHQIGVHVLPIID--
AT1G60095  VVDG----SES-------EVIRMLRFKTNL-RT------SQLFGHETT---PSFILEK-ECHKIVGFHGKIGKM-LHQIGVNVLPITD--
AT1G60110  VVDG----SES-------EVIRMLRFKTNM-RT------SQLFGHETT---SNFTLQK-ECHKIVGFHGKIGEM-LHQIGVHVLPITD--
AT3G16460  AIFG----NEP--------IVNMLRFTTNK-RV------SIPFGIGAG---TAFEFKK-DGQKIVGFHGRAGDL-LHKFGVHVAPITK--
AT1G52030  KIFG----VEA-------EVVTSLTFKTNK-RT------SQPFGMTAG---EHFELNE-DGYKIVGFHGKAGDL-VHQIGVHAVPIFTNY
AT1G52040  KIFG----VEA-------EVVTSLMFKTNK-RT------SQPFGMTAG---EHFELKE-DGYKVVGFHGKAGDL-VHQIGVHIVPIFTNY
AT3G21380  KIFG----TEG-------EIITMLRFTTNK-RT------SPPFGLEGA---KSVLLKE-DGHKIVGFHGKAGADIIHQVGVHVKPISK--
AT3G16430  KIFG----SDG-------SVITMLRFKTNK-QT------SPPFGLEAG---TAFELKE-EGHKIVGFHGRADAL-LHKIGVHVRPVSN--
AT3G16420  KIFG----SDG-------SVITMLRFKTNK-QT------SPPFGLEAG---TVFELKE-EGHKIVGFHGRADVL-LHKIGVHVRPLSN--
AT3G16440  KIFG----FET-------EVINMLRFKTNK-KT------SPPFGIEAG---TAFELKE-EGCKIVGFHGKVSAV-LHQFGVHILPVTN--
AT3G16450  KIFG----SDG-------LIITMLRFKTNK-QT------SAPFGLEAG---TAFELKE-EGHKIVGFHGKASEL-LHQFGVHVMPLTN--
AT1G52100  ---------------------------------------------------LNWTIR------------------VSTLQLWKVVTIK--
AT5G35950  KVMG----AET-------GVLTMLRFKTNI-RI------SPSFGLKAG---FNFVLEK-EGHKINGFHGKSSSM-LHQIGIHVIPITE--
AT5G35940  KVMG----AET-------GVITMLRFKTNK-RT------SPPFGLEAG---VNFVLQK-ESHKITGFHGKSSTM-LHQIGVHVVPITE--
AT1G58160  ---------------------------------------SPAK-----------------------------------------------
AT1G57570  KVNG----TEV-------EVITMLRIQTNK-RT------SIPVGCESN---SSFVLKK-EGYKIVGFHGKASNM-INQLGVHVVPLTE--
AT1G61230  KGIE----GDV-------ESITMLKFKTNK-RT------SISFGFESS---SSFLLEK-EGFKIVGFHGKASNM-IHQLGVHVIPITH--
AT1G52050  ------------------------------------------------------------------------------------------
AT1G52060  ------------------------------------------------------------------------------------------
AT1G52070  -------------------------------PS---------------------------------------------------------
AT2G25980  KVFG----SES-------SVIVMLKFKTNK-RT------SPPYGMDAG---VSFILGK-EGHKVVGFHGKASPE-LYQIGVTVAPITK--
AT5G28520  KTSG----NER-------GVITRLRFTTNK-QT------FRPVGLEST---TSFSLGK-EGYKIVGFHGNSSTDKLHQLGVYVVPITRE-
AT1G33790  KIFG----SGG-------GVITMLKFKTNK-RT------SPPFGLETT---SNFVLGK-EGYKIVGFHGTSSHE-LHQLGVYVMPI----
AT1G05760  ------------------TVLPKIDTGNAE-ET--------------------------ESKIVLG------------------------
AT1G05770  -----------------------------------------------------------ESNIVLG------------------------
AT2G43730  ------------------------------------------------------------------------------------------
AT2G43740  PNFE--------------DKISLYQSSDRLARS------TNTRTLE-------YQIPEFLDVNPIGRKSKLKYGIFSKLARLFRNLLD--
```

```
                1360
          ....|....|....|..
AT3G59590  SFPNHSPVFNY------
AT3G59610  -----------------
AT1G19715  KSYYSRASCFKLF----
AT3G59620  -----------------
AT1G73040  -----------------
AT5G46000  -----------------
AT2G33070  -----------------
AT3G16390  -----------------
AT3G16410  -----------------
AT3G16400  -----------------
AT1G52000  -----------------
AT2G39330  -----------------
AT2G39310  -----------------
AT3G16470  -----------------
AT5G38540  VS---------------
AT5G38550  I----------------
AT1G52120  EAGRGAIHTSW------
AT1G52130  DGKALEEQGGDGGLGGV
AT1G52110  -----------------
AT5G49850  -----------------
AT5G49870  -----------------
AT5G49860  -----------------
AT1G60130  -----------------
AT1G60095  -----------------
AT1G60110  -----------------
AT3G16460  -----------------
AT1G52030  RCVF-------------
AT1G52040  RVAI-------------
AT3G21380  -----------------
AT3G16430  -----------------
AT3G16420  -----------------
AT3G16440  -----------------
AT3G16450  -----------------
AT1G52100  -----------------
AT5G35950  -----------------
AT5G35940  -----------------
AT1G58160  -----------------
AT1G57570  -----------------
AT1G61230  -----------------
AT1G52050  -----------------
AT1G52060  -----------------
AT1G52070  -----------------
AT2G25980  -----------------
AT5G28520  -----------------
AT1G33790  -----------------
AT1G05760  -----------------
AT1G05770  -----------------
AT2G43730  -----------------
AT2G43740  -----------------
```
