# Supplementary material for: Duplicate Gene Divergence by Changes in MicroRNA Binding Sites in Arabidopsis and Brassica
Source: Genome Biol Evol. 2015 Feb 2;7(3):646–55. doi: 10.1093/gbe/evv023 (PMC5322543; doi:10.1093/gbe/evv023)
Supplement: Supplementary Data [file supp_evv023_New_Microsoft_Office_Word_Document.docx]

**Supplementary figures and tables**

**Figure S1:** Proportion of duplicates and singletons in the targets identified by individual prediction programs. A. Results based on psRNAtarget. B. Results based on TAPIR. C. Results based on UEA sRNA. The parts of the pie chart representing duplicates and singletons are colored in deep grey and light grey, respectively.

**Figure S2**: Multiple sequence alignment of jacalin domain containing family in *Arabidopsis thaliana*.

**Table S1:** Lists of duplicates, singletons and duplicate gene pairs in *Arabidopsis thaliana* used in this study. Also shown are the results of the proportion of duplicated genes vs. singletons as microRNA targets using E-value cutoffs of 1e-20 and 1e-30 along with at least 50% sequence coverage.

**Table S2:** MicroRNA binding site prediction dataset and the experimental dataset of *Arabidopsis thaliana*. The microRNA-target interactions on the list of the prediction dataset are those identified by at least two out of three prediction tools. The microRNA-target interactions on the list of the experimental dataset are manually collected from the combination of multiple publications and public databases.

**Table S3:** Lists of paralog pairs with the same and divergent microRNA binding sites for both the microRNA binding site prediction dataset and the experimental dataset in *Arabidopsis thaliana*. Whole genome duplicates, tandem duplicates and other types of duplicates are colored yellow, green and light blue, respectively.

**Table S4:** Lists of young and ancient microRNAs in *Arabidopsis thaliana* and the plant species whose nuclear genomes were searched to identify young and ancient microRNAs. Young microRNAs are defined as those with homologs only found in the *Arabidopsis* genus (*Arabidopsis thaliana* and *Arabidopsis lyrata*). Ancient microRNAs are those with homologs found outside of the *Arabidopsis* genus (see Materials and Methods).

**Table S5:** List of pairs/triplets of genes with the same and divergent microRNA binding sites patterns in *Brassica rapa*. LF, MF1 and MF2 indicate three different subgenomes derived from the whole genome triplication event, respectively. The symbol of ‘-’ represents the absence of a clearly detectable homeolog. The syntenic gene information of three subgenomes is obtained from ([Cheng et al. 2012](#_ENREF_15))
